# Activation and Fixation of Atmospheric CO_2_ through a 1,2,3‐Triazole‐Based Mesoionic Carbene–Borane Adduct

**DOI:** 10.1002/chem.202403942

**Published:** 2025-03-30

**Authors:** Maren Neubrand, Jessica Stubbe, Richard Rudolf, Robert R. M. Walter, Maite Nößler, Biprajit Sarkar

**Affiliations:** ^1^ Institut für Anorganische Chemie Universität Stuttgart Pfaffenwaldring 55 Stuttgart 70569 Germany; ^2^ Institut für Chemie und Biochemie, Anorganische Chemie Freie Universität Berlin Fabeckstraße 34–36. Berlin 14195 Germany

**Keywords:** boranes, CO_2_ capture, CO_2_ fixation, formic acid, mesoionic carbenes

## Abstract

Capturing atmospheric CO_2_ and converting it to valuable chemicals are important goals in contemporary science. We present here a simple, transition metal‐free triazolylidene–borane adduct that can capture atmospheric CO_2_ and convert it to formate. Several key intermediates were isolated and characterized by a combination of multinuclear NMR spectroscopy, IR spectroscopy and single crystal X‐ray diffraction. A first closed cycle for the conversion of CO_2_ to formic acid by using the aforementioned triazolylidene–borane compound is presented as well.

## Graphical abstract

A triazole‐based mesoionic carbene (MIC)–borane compound captures atmospheric CO_2_ and converts it into formate. The rate and product formation of CO_2_ reduction is seen to be strongly dependent on the CO_2_ source with both formate and bicarbonate being produced with the same MIC–borane compound. A battery of spectroscopic methods was used to investigate the intermediates and understand the underlying mechanism.

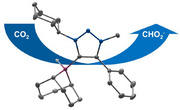

## 1 Introduction

Climate change is an undeniable fact, and the escalating level of atmospheric carbon dioxide is one of the most pressing environmental concerns of our age. The environmental consequences are the basis for political initiatives on a global scale, which are driven by the aim of reducing the emission of greenhouse gases, most notably carbon dioxide.^[^ ^1^ ^]^ Technologies exist that are able to trap the CO_2_ from the flue gas of major emitters; however, the price of carbon dioxide is too low to be a motivation for industries to increase their efforts in recovering the greenhouse gas.^[^ ^2^ ^]^ The utilization of CO_2_ as a C_1_ feedstock for the generation of industrially relevant chemicals is certainly an interesting, different approach.^[^ ^3^ ^]^ CO_2_ is an attractive renewable C_1_ source, which can lead to fuel‐related products such as methane, methanol, and formic acid.^[^ ^4^ ^]^ Those approaches would not only reduce the carbon dioxide emission through carbon capture, but the costs of the sequestration could also be compensated by the production of chemicals that are in global demand.^[^ ^5^ ^]^ Most of the liquid fuels used are based on fossil fuels, which again generate new CO_2_ in the atmosphere. Indeed, by utilization of existing CO_2_ for the generation of liquid fuels, the carbon footprint could be significantly reduced. Methanol is thereby one of the most promising energy vectors, since it could replace liquid fuels in modern technologies.^[^ ^4^ ^]^

**Figure 1:**
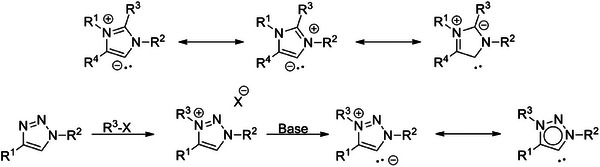
Resonance structures of a generic imidazole based MIC (top) and a generic triazole based MIC (with an exemplary resonance formula), obtained by alkylation of the triazole and deprotonation of the triazolium salt (bottom).

In the past years, several reports based on the reduction of CO_2_ through various transition metal compounds,^[^ ^6^, ^7^, ^8^, ^9^, ^10^, ^11^, ^12^ ^]^ metal‐free catalysts^[^ ^13^, ^14^, ^15^, ^16^, ^17^, ^18^ ^]^ or direct reduction with NaBH_4_ without catalysts^[^ ^19^ ^]^ have been published. But it is still a problem to capture the CO_2_ present in the atmosphere due to its low concentration and, furthermore, to reduce it to obtain fuels under ambient conditions. The first report on transition metal‐free CO_2_ capture from air, including the consecutive reduction of the CO_2_ to produce a methanol precursor, which can be easily converted into methanol under ambient conditions, was made by the group of Mandal in 2019.^[^ ^20^ ^]^ They reported an imidazole‐based mesoionic carbene (MIC) (Figure 1) supported 9‐BBN adduct, which is able to perform the described conversion, including a thorough investigation of the mechanistic pathway. In recent years, the combination of a B‐center together with carbenes or other main group components has turned out to be an attractive strategy for activating and converting inert bonds in small molecules.^[^ ^21^, ^22^, ^23^, ^24^, ^25^, ^26^, ^27^, ^28^ ^]^

Since the first report on MIC of the 1,2,3‐triazol‐5‐ylidene (Figure 1) type more than 15 years back, the chemistry of this compound class has been dominated by transition metals^[^ ^29^, ^30^, ^31^, ^32^ ^]^; prominent examples include applications in homogeneous catalysis, electrocatalysis, photochemistry/photophysics, and in the stabilization of unusual metal oxidation states.^[^ ^29^, ^30^, ^31^, ^32^, ^33^, ^34^, ^35^ ^]^ In addition, a few reports have appeared either on the transformations of MICs into organic redox systems,^[^ ^36^ ^]^ or on the combination of MICs with main group element fragments.^[^ ^29^, ^37^, ^38^, ^39^ ^]^ In the following, we report the synthesis and characterization of a 1,2,3‐triazol‐5‐ylidene‐based MIC‐supported 9‐BBN adduct. This MIC–borane adduct can capture atmospheric CO_2_, and convert it to formic acid. Investigations of a few key intermediates via multi‐nuclear NMR spectroscopy, IR spectroscopy, and single‐crystal x‐ray diffraction allow us to postulate a plausible reaction mechanism. In addition, a first closed cycle for this process is presented as well.

## 2 Results and Discussion

### 2.1 Synthesis and Characterization of the MIC–Borane Adduct

**Scheme 1:**
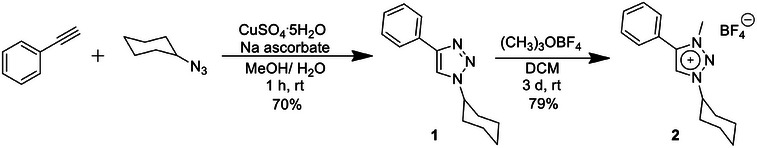
Synthesis of the triazole **1** and the triazolium salt **2**.

Our research started with the synthesis of the triazolium salt **2** as a precursor for the desired MIC‐9‐BBN adduct. **2** was generated through methylation of the 1,2,3‐triazole **1** using Meerwein's reagent, and the triazole **1** itself was constructed by the cycloaddition of cyclohexyl azide and phenyl alkyne under standard click conditions (Scheme 1).

**Scheme 2:**
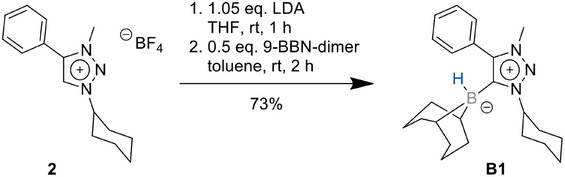
Synthesis of the MIC–borane adduct **B1**.

With the aim of generating a MIC‐9‐BBN (9‐BBN═(9‐borabicyclo[3.3.1]nonane)) adduct, **2** was deprotonated with LDA at room temperature in the presence of the 9‐BBN dimer (Scheme 2).

**Figure 2:**
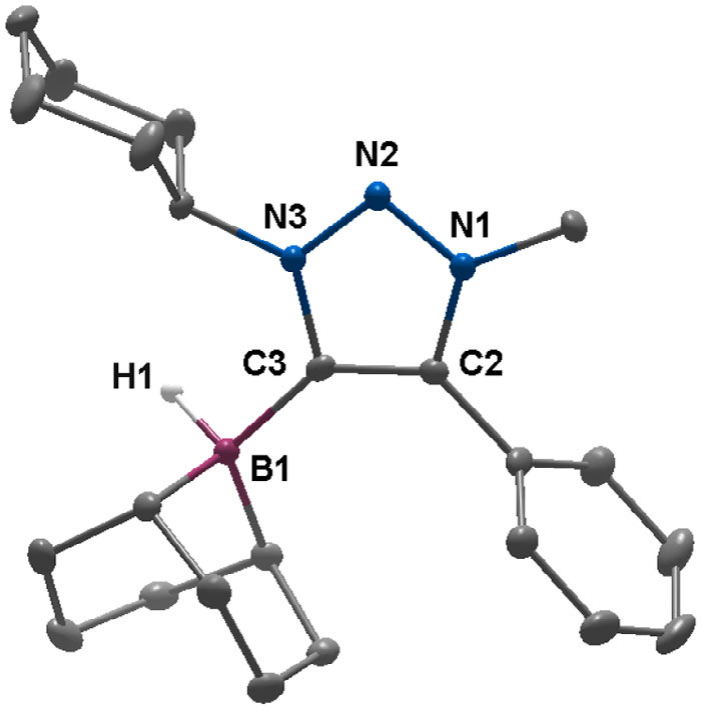
ORTEP representation of **B1**: ellipsoids drawn at 50% probability. Solvent molecules and H‐atoms omitted for clarity. (For selected bond lengths and angles see supporting information Table S1).

The reaction progress was controlled via ^1^H NMR spectroscopy, in which the disappearance of the triazolium‐*H* signal at 8.58 ppm indicates the clean deprotonation of the former triazolium salt. The nucleophilic attack of the in situ generated 1,2,3‐triazol‐5‐ylidene to the 9‐BBN dimer led to the formation and isolation of the 1,2,3‐triazol‐5‐ylidene‐based MIC‐9‐BBN adduct **B1** in 73% yield. Single crystals could be obtained by the slow diffusion of *n*‐hexane into a toluene solution of **B1** under inert conditions. **B1** displays a signal at −17.2 ppm in the ^11^B spectrum. In the solid‐state molecular structure of **B1,** the bond lengths within the triazolylidene moiety are in accordance with values previously reported in the literature (Figure 2).^[^ ^30^ ^]^ The distance between the carbene C3 and the boron B1 is 1.634(2) Å and is in the same range as reported earlier for related compounds.^[^ ^20^ ^]^

The electrochemical properties of **B1** were investigated through cyclic voltammetric measurements (CV) in a MeCN/0.1 м NBu_4_PF_6_ solution, (Figure S7). The CV shows a single reduction and two oxidation waves, which are all irreversible. The irreversibility is not influenced by increasing the scan rates of the measurements.

### 2.2 Reaction of B1 with Atmospheric CO_2_

**Scheme 3:**
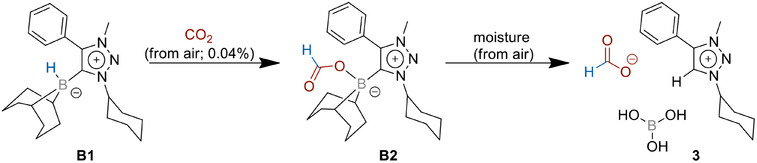
Reaction of **B1** with atmospheric CO_2_.

We were interested in the capability of **B1** in capturing CO_2_ from air; therefore, a solution of **B1** was stirred in benzene under ambient air overnight. The resulting ^1^H NMR spectrum shows a crude reaction mixture, including the intact **B1** and an additional product mixture, which includes the compounds **B2** and **3** (Scheme 3), analogous to the observations made by Mandal and co‐workers.^[^ ^17^ ^]^ In ^1^H NMR spectra recorded in CD_3_CN, characteristic signals of all compounds can be found between 4.6 and 5.3 ppm, corresponding to the cyclohexyl‐C*H* attached to the triazolium unit. These signals are low‐field shifted due to the positive charge of the close triazolium moiety and integrate to one proton. Considering these signals, a clear distinction of present species or at least the number of species in the reaction mixture can be made. The crude NMR (see Figure S18) shows three of those characteristic multiplets, therefore corresponding to at least three different species in the mixture. The low‐field region of the spectra shows two sharp and one broadened singlet at 8.74, 8.62, and 8.26 ppm. In a mixture of **B2** and **3**, one would expect a signal caused by the attached formate in **B2** and two singlets corresponding to the formate anion and the newly formed triazolium‐*H* in **3**. Crystallization attempts in an acetonitrile/diethyl ether mixture of the crude reaction mixture led to the formation of single crystals. Unfortunately, we were not able to collect structural data of the crystal of sufficient quality for the discussion of bond lengths and bond angles. However, the connectivity in the molecule is observed and presents one CO_2_ molecule fixed as formate to the MIC‐9‐BBN adduct, leading to the compound **B2** (Figure S4). These NMR and crystallographic results thus clearly show that the MIC–borane adduct **B1** can bind atmospheric CO_2_ and convert it to formate. Unfortunately, the compounds **B2** and **3** generated through this method could not be separated and isolated in bulk. We therefore followed two alternative strategies to achieve a full characterization of the products and to establish a first closed cycle for CO_2_ reduction with respect to **B1**.

### 2.3 Reaction of B1 with Dry Ice

**Scheme 4:**
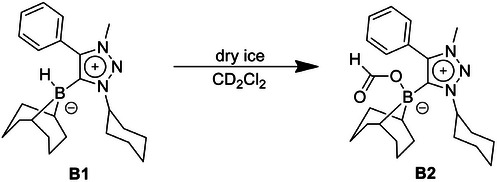
Reaction of **B1** with dry ice for the formation of the formate complex **B2**.

The reaction of **B1** with dry ice as a CO_2_ source led to the clean formation of **B2** (Scheme 4).

**Figure 3:**
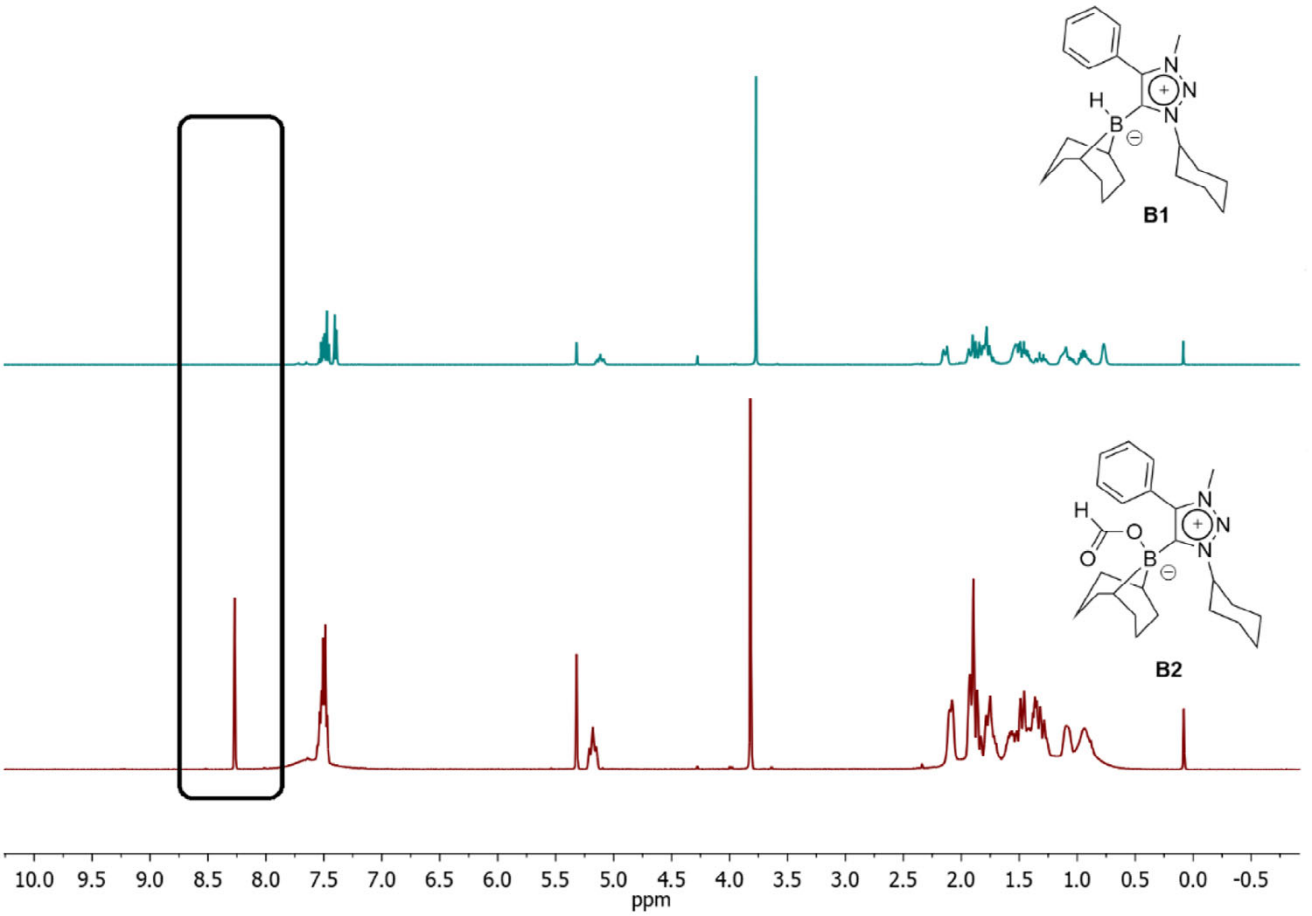
^1^H NMR spectra of **B1** (top) and **B2** (bottom) in CD_2_Cl_2_. The black box on the left‐hand side shows the appearance of the signal corresponding to the formate proton in **B2**.

**Figure 4:**
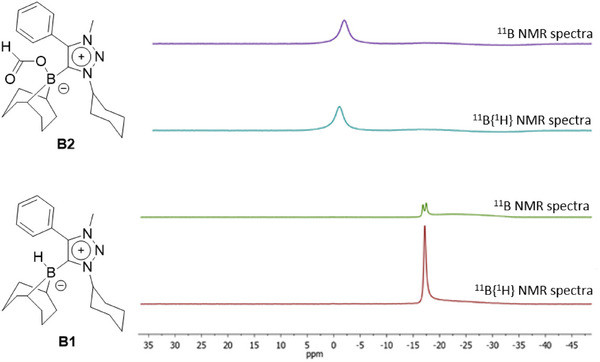
^11^B NMR spectra of **B1** (bottom) and **B2** (top) measured in CD_2_Cl_2_.

**Figure 5:**
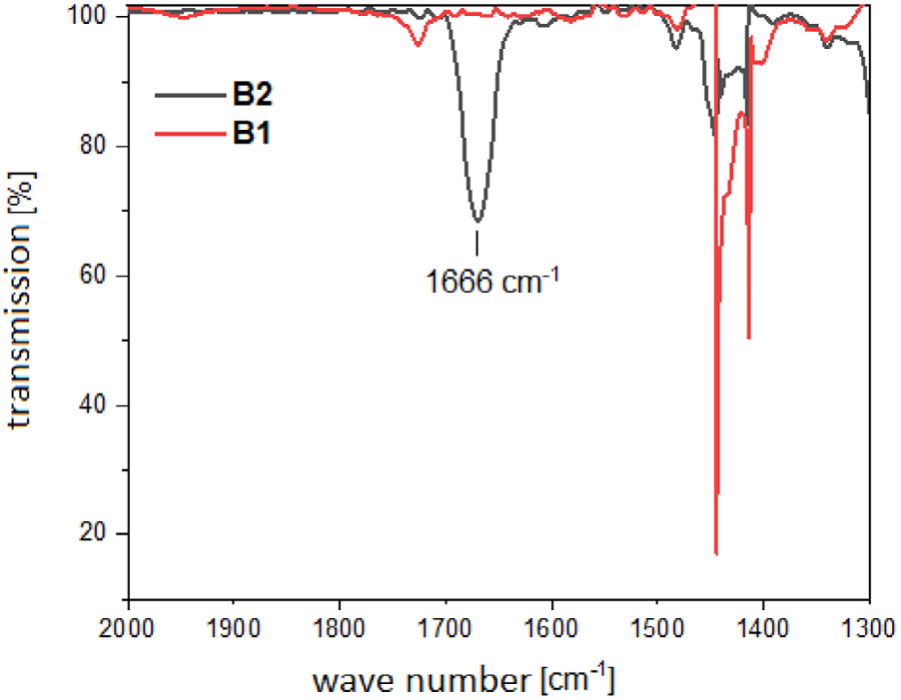
IR spectra of **B1** and **B2** in CH_2_Cl_2_.

The formation of **B2** was confirmed by several spectroscopic methods. In the ^1^H NMR spectrum a new singlet appears at 8.27 ppm, which can be assigned to the formate‐proton (Figure 3). The signals obtained for **B2** match with those observed in the crude reaction mixture of **B1** with atmospheric CO_2_ (Figure S18). In the ^11^B NMR spectrum, **B2** displays a singlet at −1.46 ppm which is a completely different chemical shift in comparison to **B1** (Figure 4). In addition, in the ^1^H‐coupled ^11^B spectrum, **B1** shows a doublet, whereas **B2** displays only a singlet, indicating the loss of the B─H proton. Furthermore, a strong peak is observed at 1666 cm^−1^ in the IR spectrum of **B2** (Figure 5). This peak can be assigned to the carbonyl group of the formate in **B2**.

**Figure 6:**
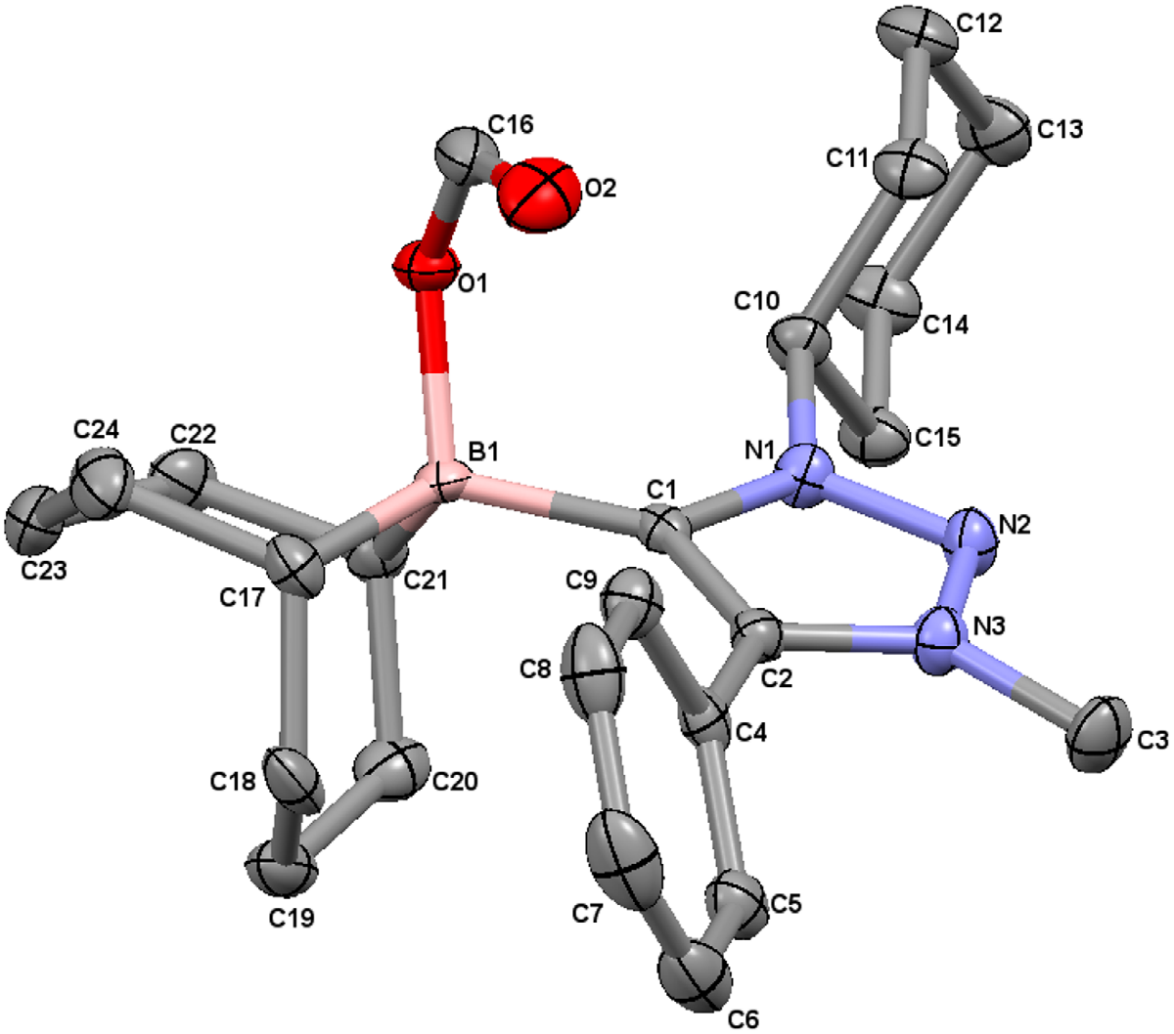
ORTEP representation of **B2**. Ellipsoids are drawn at 50% probability. Solvent molecules and H‐atoms have been omitted for clarity. (For selected bond lengths and angles see Table S3).

The final unequivocal proof for the formation of **B2** came from its single crystal X‐ray diffraction analysis (Figure 6). As can be seen from the molecular structure in the crystal, the formate is bound to the boron center. The C1─B1 bond length between the MIC─C and the boron center is 1.644(6) Å and is in the same range as that observed for **B1**. The O1─C16 and the O2─C16 bond lengths of formate in **B2** are 1.298(5) and 1.208(5), respectively, indicating as expected one shorter and one longer C─O bond. The B1─O1 bond distance is 1.551(6) Å. This structure is also identical in terms of connectivity to the structure obtained from the reaction of **B1** with atmospheric CO_2_ (Figure S4).

### 2.4 Reaction of B1 with other CO_2_ Sources

**Scheme 5:**
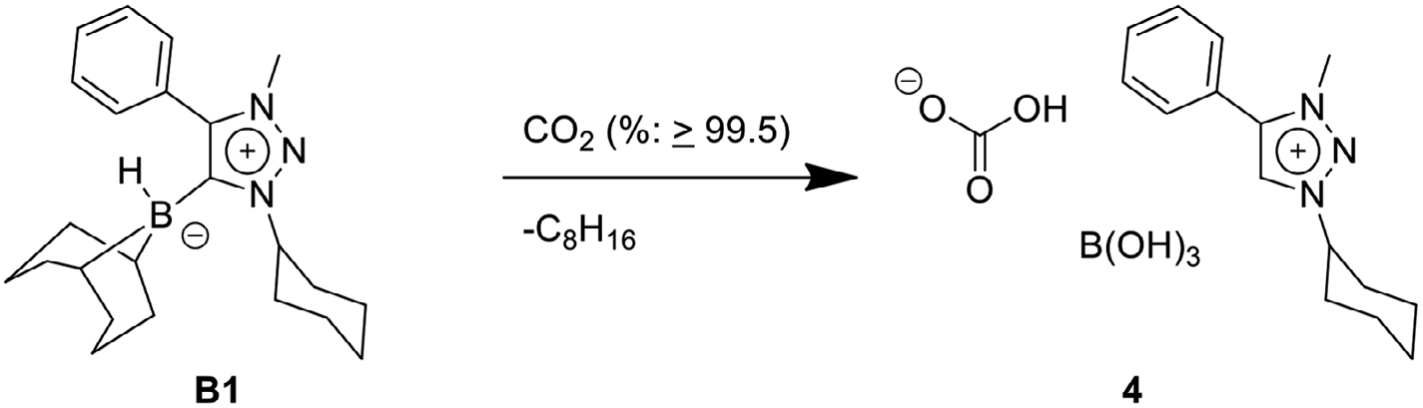
Reaction of **B1** with “pure” CO_2_ gas dissolved in benzene.

**Figure 7:**
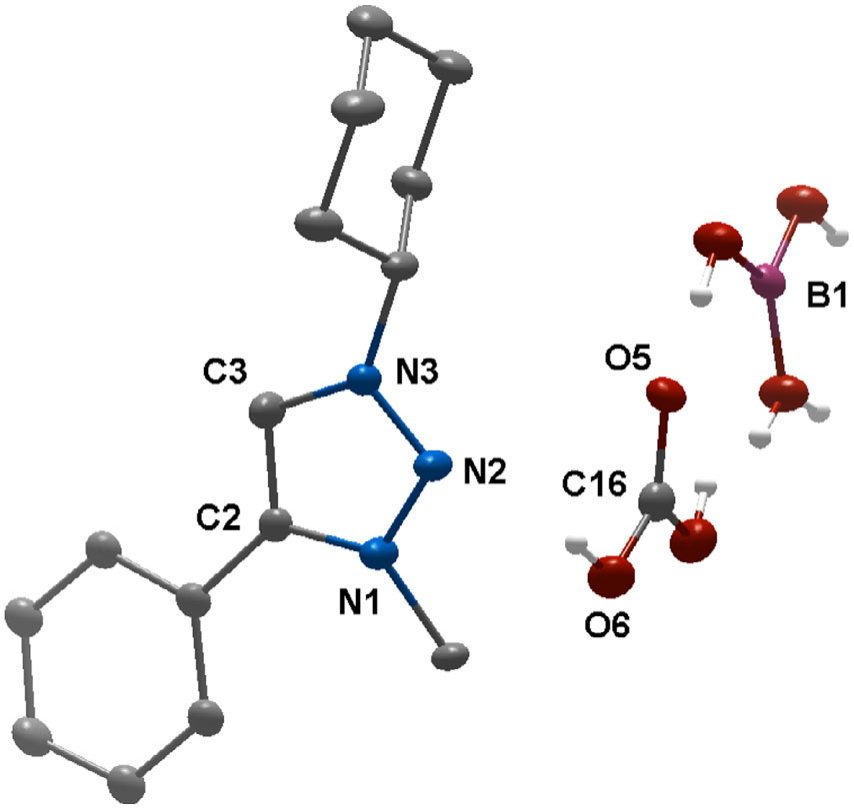
ORTEP representation of **4**. Ellipsoids drawn at 50% probability. Solvent molecules and H‐atoms omitted for clarity. (For selected bond lengths and angles see Table S2).

In another attempt, CO_2_ gas (purity % ≥ 99.5) was bubbled through a solution of **B1** in benzene (Scheme 5). In the ^1^H spectrum of the product **4** two singlets are observed at 9.31 and 8.47 ppm, and these can be assigned to the O─H proton of bicarbonate and to the C─H proton of the triazolium salt (Figure S15). In addition, a signal at +18.8 ppm in the ^11^B NMR spectrum of **4** confirms the presence of boronic acid. Gratifyingly, we were also able to obtain single crystals of **4**, suitable for X‐ray diffraction studies. The data clearly show the formation of an unprecedented triazolium salt of bicarbonate together with boronic acid (Figure 7). In the solid‐state molecular structure of **4** all bond lengths within the triazolium moiety are in accordance with values previously reported in the literature.^[^ ^21^ ^]^ Due to the quality of the crystal data and the symmetry, two H's of the CO_3_ unit and two H's of the BO_3_ have a reduced occupation of one and a half. Intriguingly, while the reaction of **B1** with atmospheric CO_2_ leads eventually to the formation of triazolium formate together with boronic acid (Scheme 3), the reaction of **B1** with “pure” CO_2_ dissolved in a solvent leads to the formation of triazolium bicarbonate and boronic acid (Scheme 5). These results thus show the sensitivity of the reactivity of **B1** towards the concentration of CO_2_ and/or pH of the solution.

### 2.5 Generation of a Closed Cycle for formate Formation

**Scheme 6:**
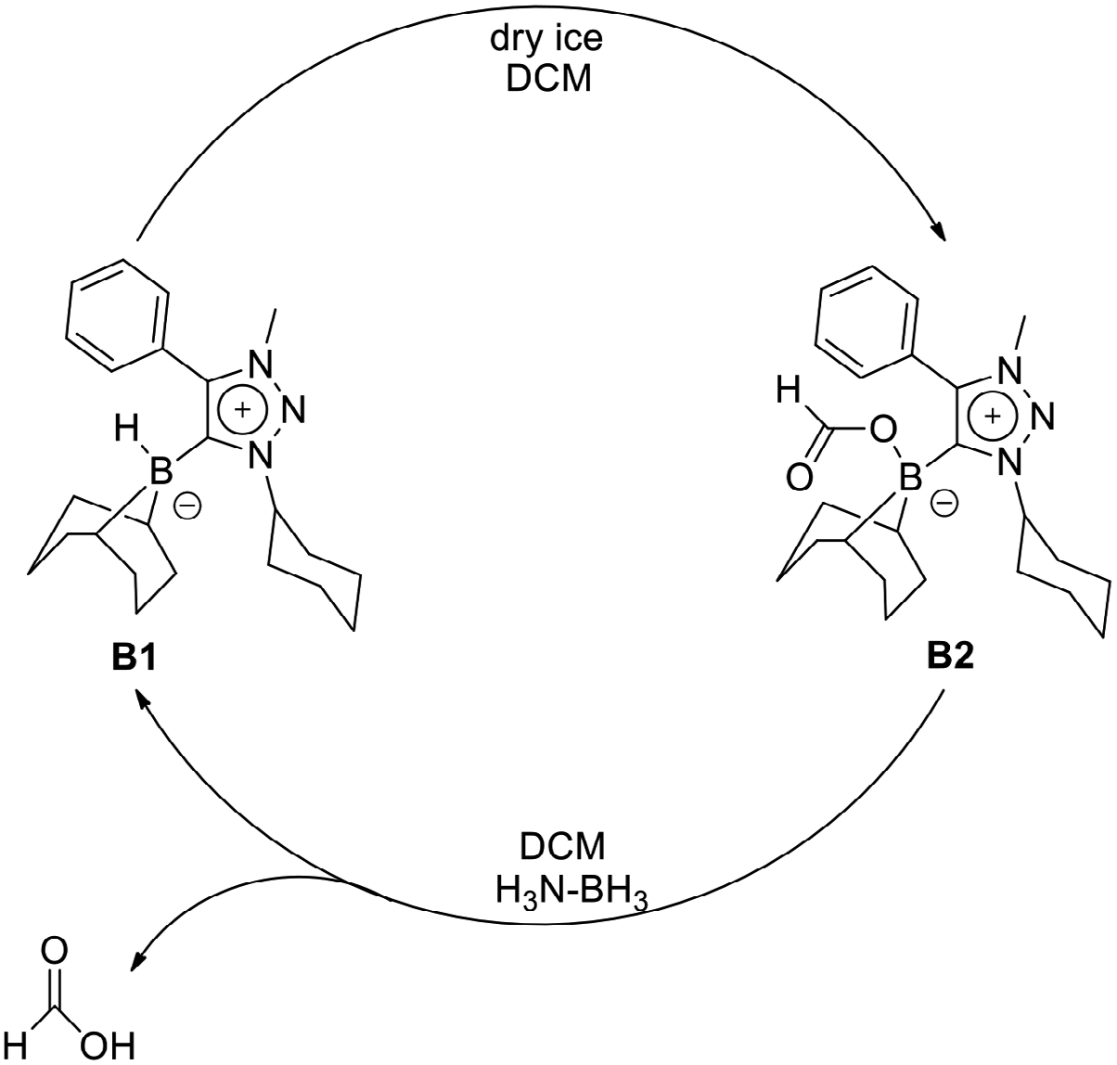
Conversion of **B1** to **B2** through activation of CO_2_, and the conversion of **B2** back to **B1** by using ammonia borane as a proton and a hydride source.

After having all these results in hand, we looked for a way of regenerating **B1** from **B2** coupled with the formation of formic acid. Gratifyingly, as described above, **B1** can be converted to **B2** by using dry ice as a CO_2_ source, and **B2** can be converted back to **B1** by using ammonia borane as a proton and a hydride donor to regenerate **B1** through the simultaneous formation of formic acid (Scheme 6, Figure S23,24). The formic acid was detected either through a direct GC analysis or by precipitating it as sodium formate (see Supporting Information).

## 3 Conclusions

Summarizing, we have presented here a 1,2,3‐triazol‐5‐ylidene‐based MIC‐supported 9‐BBN adduct **B1**. The adduct is capable of capturing and reducing CO_2_ from air. The greenhouse gas binds to the adduct as formate, which could then be further utilized after cleavage as a formate counter‐anion for the generated triazolium salt and boronic acid. The reaction of the MIC–borane adduct with CO_2_ was found to be strongly dependent on the source of CO_2_, as atmospheric CO_2_ led to the formation of a formate triazolium salt, whereas “pure” CO_2_ resulted in the formation of a triazolium bicarbonate. By using dry ice, we were able to establish a closed cycle in which the MIC–borane adduct, activates and binds CO_2_ to form a compound in which the resulting formate is bound to the boron center. That compound could be reacted with ammonia borane to produce formate and regenerate the MIC–borane adduct thus closing the cycle. Besides showcasing the ability of MIC–boranes for CO_2_ capture and fixation, our results in general point to the hidden potential of triazolylidene adducts with main group fragments for activating and converting inert small molecules.

### 3.1 X‐ray Diffraction

Deposition Number(s) “https://www.ccdc.cam.ac.uk/services/structures?id” “https://doi.org/10.1002/chem.202403942” 2 052 661 (for **B1**), 2 379 971 (for **B2**), 2 052 662 (for **4**) contain(s) the supplementary crystallographic data for this paper. These data are provided free of charge by the joint Cambridge Crystallographic Data Centre and Fachinformationszentrum Karlsruhe “https://www.ccdc.cam.ac.uk/structures” Access Structures service.

**Electronic supplementary information (ESI) available**: Synthetic procedures, NMR spectra, HPLC data, X‐ray crystallographic data, and electrochemical data.

## Conflict of Interests

The authors declare no conflicts of interest.

## Supporting information



Supporting Information
