# Supplementary material for: Activation and Fixation of Atmospheric CO2 through a 1,2,3‐Triazole‐Based Mesoionic Carbene–Borane Adduct
Source: Chemistry. 2025 Mar 30;31(25):e202403942. doi: 10.1002/chem.202403942 (PMC12057606; doi:10.1002/chem.202403942)
Supplement: Supplementary file 1 — Supporting Information [file CHEM-31-e202403942-s001.docx]

**taSupporting Information**

Maren Neubrand, Jessica Stubbe, Richard Rudolf, Robert R. M. Walter, Maite Nößler, Biprajit Sarkar

[1. Instrumental 2](#_Toc172041478)

[2. Synthetic procedures 3](#_Toc172041479)

[3. HPLC measurements for the detection of formic acid 8](#_Toc172041480)

[4. Crystal structures and crystallographic data 9](#_Toc172041481)

[5. Cyclic voltammetry 13](#_Toc172041482)

[6. NMR Spectroscopy 14](#_Toc172041483)

[7. References 23](#_Toc172041484)

## Instrumental

Unless otherwise noted, all reactions were performed using standard Schlenk-line techniques under an inter atmosphere of argon (Linde, Argon 4.8, purity ≥ 99.998) or an MBraun glove box fitted with a gas purification and recirculation unit. Commercially available chemicals were used without further purification. THF, toluene, benzene and *n*-hexane were dried and distilled from sodium, acetonitrile from phosphorus pentoxide. Other solvents were available from MBRAUN MB-SPS-800 solvent system and additionally degassed using standard techniques.

^1^H NMR, ^13^C NMR and ^11^B NMR were recorded on Joel ECS 400 and 400R spectrometer.

Chemical shifts are reported in ppm (relative to the TMS signal) with reference to the residual solvent peaks.^[1]^ Multiplets are reported as follows: singlet (s), duplet (d), triplet (t), quartet (q), quintet (quint), and combinations thereof. NMRs under the exclusion of air were conducted in J. Young’s NMR tubes oven dried and flushed with argon prior to use.

Cyclic voltammograms were recorded with a PAR VersaStat 4 potentiostat (Ametek) or Palm Sens 4 potentiostat by working in anhydrous and degassed acetonitrile with 0.1 м NBu_4_PF_6_ (dried, > 99.0%, electrochemical grade, Fluka) as supporting electrolyte. Concentrations of the compounds were about 1·10^-4^ м. A three-electrode setup was used with a glassy carbon or gold working electrode, a coiled platinum wire as counter electrode, and a coiled silver wire as a pseudo-reference electrode. The ferrocene/ferrocenium couple was used as internal reference.

X-ray data were collected on a Bruker Smart AXS or Bruker D8 Venture systems at 140(2) K or 100(2) K, respectively, using graphite-monochromated Mo_Κα_ radiation (λ_α_= 0.71073 Å). The strategy for the data collection was evaluated by using the Smart software. The data were collected by the standard omega scan or omega + phi scan techniques, and were scaled and reduced using Saint+ and SADABS software. The structures were solved by direct methods using SHELXS-97 or intrinsic phasing using SHELXL-2014/7 and refined by full matrix least-squares, refining on F^2^. Non-hydrogen atoms were refined anisotropically.^[2-8]^ Structures were solved using SHELXS-97^[4]^ and the software OLEX2,^[9]^ while refinement was carried out on F2 against all independent reflections by the full matrix least-squares method using the SHELXL-97 program. All non-hydrogen atoms were refined using anisotropic thermal parameters.

## Synthetic procedures

1-Cyclohexyl-4-phenyl-1*H*-1,2,3-triazole **1**

Cyclohexyl azide was synthesized according to a literature procedure.^[10]^

Under ambient conditions, phenylacetylene (3.4 g, 15.0 mmol) and cyclohexyl azide (1.9 g, 15.0 mmol) were stirred in a methanol (60 mL) / water (10 mL) mixture at room temperature. Sodium ascorbate (1.9 g, 6.0 mmol) was added and the mixture kept in an ultrasonic bath for 15 min. Then, copper sulfate pentahydrate (0.8 g, 9.0 mmol) was added and the mixture kept in an ultrasonic bath for additional 60 min. The mixture was quenched with aqueous Na_2_EDTA/NH_4_OH solution and extracted with dichloromethane. The combined organic layers were dried with Na_2_SO_4_. Removal of the volatiles gave a yellow oil, which was redissolved in small amounts of dichloromethane and added into an excess of *n*-hexane. The precipitant was collected by filtration as a white solid in 70% yields (2.4 g).

**^1^H NMR** (401 MHz, 21 °C, CDCl_3_): δ 7.86–7.78 (m, 2H, phenyl-*H*), 7.78 (s, 1H, triazole-*H*), 7.46–7.27 (m, 3H, phenyl-*H*), cyclohexyl-*H*: 4.47 (tt, *J* = 11.8, 3.9 Hz, 1H), 2.29–2.18 (m, 2H), 1.93 (dp, *J* = 16.4, 3.7 Hz, 2H), 1.85–1.70 (m, 3H), 1.54–1.38 (m, 2H), 1.28 (qt, *J* = 12.9, 3.6 Hz, 1H) ppm.

**^13^C NMR** (126 MHz, 27 °C, CDCl_3_): δ 147.5, 131.1, 128.9, 128.1, 125.8, 117.4, 60.3, 33.8, 25.3, 25.3 ppm.

1-Cyclohexyl-3-methyl-4-phenyl-1*H*-1,2,3-triazol-3-ium **2**

Under an inert atmosphere of argon **1** (500 mg, 2.2 mmol) and trimethyloxonium tetrafluoroborate (488 mg, 3.3 mmol) were dissolved in dry dichloromethane (20 mL). The mixture was stirred for 3 d at room temperature. The solution was quenched with methanol (5 mL) and the mixture was added to an excess of diethyl ether (150 mL) under ambient conditions. The precipitant was collected by filtration and the product was obtained as white solid in yields of 79% (573 mg).

**^1^H NMR** (500 MHz, 27 °C, CDCl_3_): δ 8.58 (s, 1H, triazole-*H*), 7.63–7.52 (m, 5H, phenyl-*H*), 4.74 (tt, *J* = 11.5, 3.8 Hz, 1H, cyclohexyl-*H*_2_), 4.25 (s, 3H, methyl-*H*_3_), 2.38–2.31 (m, 2H, cyclohexyl-*H*_2_), 1.96–1.88 (m, 4H, cyclohexyl-*H*_2_), 1.79–1.72 (m, 1H, cyclohexyl-*H*_2_), 1.56–1.43 (m, 2H, cyclohexyl-*H*_2_), 1.30 (qt, *J* = 13.2, 3.6 Hz, 1H, cyclohexyl-*H*_2_) ppm.

**^13^C NMR** (126 MHz, 25 °C, CDCl_3_): δ 131.9, 129.8, 129.6, 127.2, 65.0, 38.5, 32.6, 24.8, 24. ppm.

**Elemental analysis** calcd. (%) for C_15_H_20_BF_4_N_3_: C 54.74, H 6.12, N 12.77; found: C 54.78, H 6.15, N 12.81.

1-Cyclohexyl-3-methyl-4-phenyl-1*H*-1,2,3-triazol-3-ium-5-yl-9-borabicyclo[3.3.1]nonane **B1**

Under an inert atmosphere of argon, **2** (242.9 mg, 0.738 mmol) was dissolved in absolute THF (10 mL). Lithium diisopropylamide (0.4 mL, 1.82 M in THF, heptane, ethylbenzene) was slowly added to the solution at room temperature. After 1 h, the solvents were removed under recued pressure. In the meantime, 9-BBN-dimer (90.0 mg, 0.369 mmol) was dissolved in absolute toluene (15 mL). The solution was added to the remaining solids of the **2**(BF_4_)/LDA mixture and kept stirring for 2 h. The solution was concentrated under reduced pressure (~5 mL) and filtrated *via* a syringe filter (PTFE, 0.2 µm). Single crystals were obtained after the slow diffusion of *n*-hexane into the solution, yielding in 196.1 mg (73.1%) of the desired product **B1** as colourless needles.

**^1^H NMR** (401 MHz, 20 °C, CD_2_Cl_2_): δ = 7.56–7.43 (m, 3H), 7.42–7.36 (m, 2H), 5.10 (tt, J = 11.8, 3.7 Hz, 1H), 3.77 (s, 3H), 2.17–2.09 (m, 3H), 1.96–1.88 (m, 3H), 1.88–1.65 (m, 7H), 1.55–1.37 (m, 3H), 1.30 (qt, J = 12.9, 3.5 Hz, 1H), 1.17–0.99 (m, 3H), 0.92 (tt, J = 10.9, 5.7 Hz, 3H), 0.76 (s, 2H) ppm.

**^13^C NMR** (101 MHz, 20 °C, CD_3_CN): δ = 132.0, 130.7, 129.5, 129.1, 60.7, 37.8, 37.5, 34.3, 31.5, 26.4, 26.0, 25.8 ppm.

**^11^B NMR** (129 MHz, 20 °C, CD_2_Cl_2_): δ = –17.2 ppm.

**Elemental analysis** calcd. (%) for C_23_H_34_BN_3_: C 76.03, H 9.43, N 11.56; found: C 75.86, H 9.01, N 12.01.

Reaction of **B1** with atmospheric CO_2_.

Under ambient conditions, **B1** (30.0 mg, 0.083 mmol) was dissolved in benzene (5 mL) and stirred overnight. The solvent was removed under reduced pressure. The remaining solids were redissolved in CH_3_CN and colourless single crystals were obtained by the slow diffusion of diethyl ether into the solution. Compound **B2** could not be isolated in mass and further characterised using this strategy.

1-Cyclohexyl-3-methyl-4-phenyl-1*H*-1,2,3-triazol-3-ium bicarbonate boric acid **4**

Under an inert atmosphere of argon, **B1** (38.4 mg, 0.106 mmol) was dissolved in absolute benzene (10 mL) and CO_2_ gas (purity % ≥ 99.5) was bubbled through the solution for 5 min. The reaction was allowed to stir under an atmosphere of CO_2_ for additional 12 h. During the reaction time, a white precipitant occurs, which was filtered off. The solvent was removed under reduced pressure and the remaining solids redissolved in CH_3_CN. Single crystals were obtained by the slow diffusion of diethyl ether into the solution and yielded the product **4** as white crystals in 45% yields (17.6 mg).

**^1^H NMR** (401 MHz, 22 °C, DMSO-*d*_6_): δ = 9.31 (s, 1H), 8.47 (s, 1H), 7.81–7.72 (m, 2H), 7.72–7.60 (m, 3H), 4.82 (tt, *J* = 11.1, 3.8 Hz, 1H), 4.29 (s, 3H), 4.06 (d, *J* = 3.0 Hz, 0H), 3.60–3.50 (m, 4H), 2.29–2.19 (m, 2H), 1.94–1.79 (m, 5H), 1.79–1.63 (m, 4H), 1.63–1.46 (m, 2H), 1.46–1.19 (m, 4H) ppm.

**^13^C NMR** (101 MHz, 22 °C, DMSO-*d*_6_): δ = 165.8, 142.2, 131.5, 129.4, 129.3, 127.4, 122.87, 69.84, 63.1, 36.2, 35.4, 32.2, 31.8, 26.1, 24.3, 24.1, 20.0, 18.8 ppm.

**^11^B NMR** (129 MHz, 22 °C, DMSO-*d*_6_): δ = 18.8 ppm.

Mesoionic formate-carbene borane adduct **B2**

The reaction and work-up were carried out under argon atmosphere. The mesoionic carbene borane adduct **B1** was dissolved in CD_2_Cl_2_. Then several small pieces of dry ice were added one after the other under argon flow and stirred for 12 h. After removal of the solvent under reduced pressure, the product **B2** was obtained as a white solid with full conversion.

**^1^H NMR** (400 MHz, CD_2_Cl_2_): δ = 7.74- 7.58 (m, 1 H, Aryl-*H*), 7.58- 7.42 (m, 4 H, Aryl-H ), 5.18

(tt, *J* = 11.5, 3.6 Hz, 1H, cyclohexyl-*H*), 3.82 (s, 3H, CH_3_), 2.18-2.03 (m, 2H), 1.97-1.81 (m, 5H) 1.81-1.67 (m, 3H), 1.64-1.51 (m, 2H), 1.51-1.24 (m, 7H), 1.09 (s, 2H), 1.10-1.82 (m, 3H) ppm.

**^11^B NMR** (400 MHz, CD_2_Cl_2_): δ = -1.46 ppm.

**^13^C NMR** (126 MHz, CDCl_3_): δ = 165.1 (C-Formiat), 143.6, 130.7, 127.8, 125.4, 62.7, 37.6, 34.3, 31.3, 26.1, 25.7, 24.8, 24.1 ppm.

**HRMS (ESI):** m/z [M+H] calcd. for C_24_H_34_N_3_O_2_: 408.2821; found: 408.2826.

Reaction of **B2** with amminborane

The mesoionic formate-carbene borane adduct **B2** was filled into an NMR tube under exclusion of air and dissolved with CD_2_Cl_2_. Amminborane was then added, also under an argon atmosphere. The NMR tube was sealed in the absence of air and measured after 12 h.

**^1^H NMR** (400 MHz, CD_2_Cl_2_): δ = 7.56–7.43 (m, 3H), 7.42–7.36 (m, 2H), 5.10 (tt, J = 11.8, 3.7 Hz, 1H), 3.76 (s, 3H), 2.17–2.09 (m, 3H), 1.96–1.88 (m, 3H), 1.88–1.65 (m, 7H), 1.55–1.37 (m, 3H), 1.30 (qt, J = 12.9, 3.5 Hz, 1H), 1.17–0.99 (m, 3H), 0.92 (tt, J = 10.9, 5.7 Hz, 3H), 0.76 (s, 2H) ppm.

**^11^B NMR** (400 MHz, CD_2_Cl_2_): δ = –17.1 ppm.

Reaction of **B1** with sodium formate

Sodium hydroxide was added to the mixture of formic acid and **B1** and the precipitated solid was filtered off and D_2_O and DMSO were added for NMR analysis.

**^1^H NMR** (400 MHz, D_2_O): δ = 8.4 ppm.

## HPLC measurements for the detection of formic acid


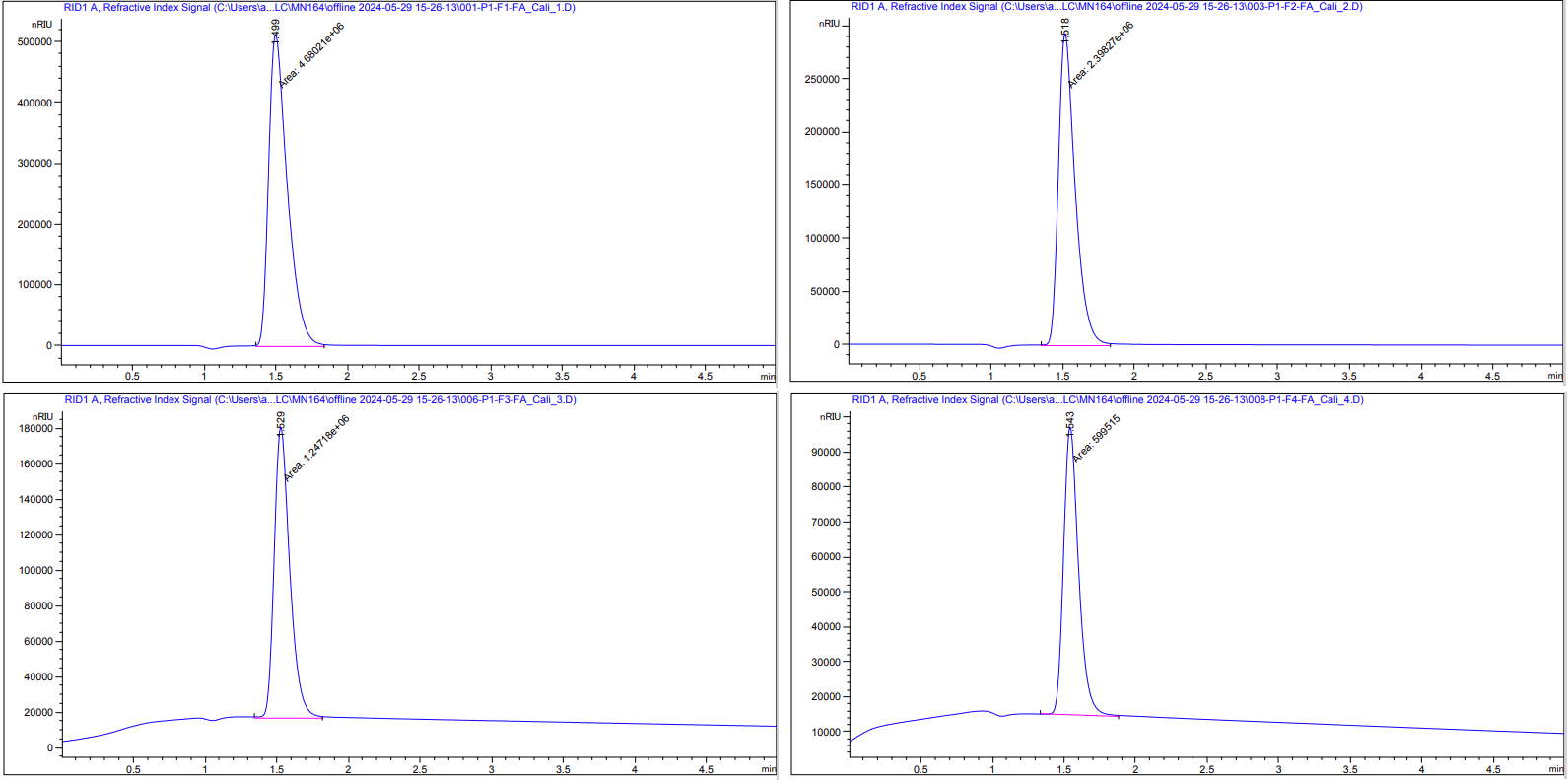


| **Figure S1:** *HPLC graphs of the calibration with DCM and formic acid.* |
| --- |


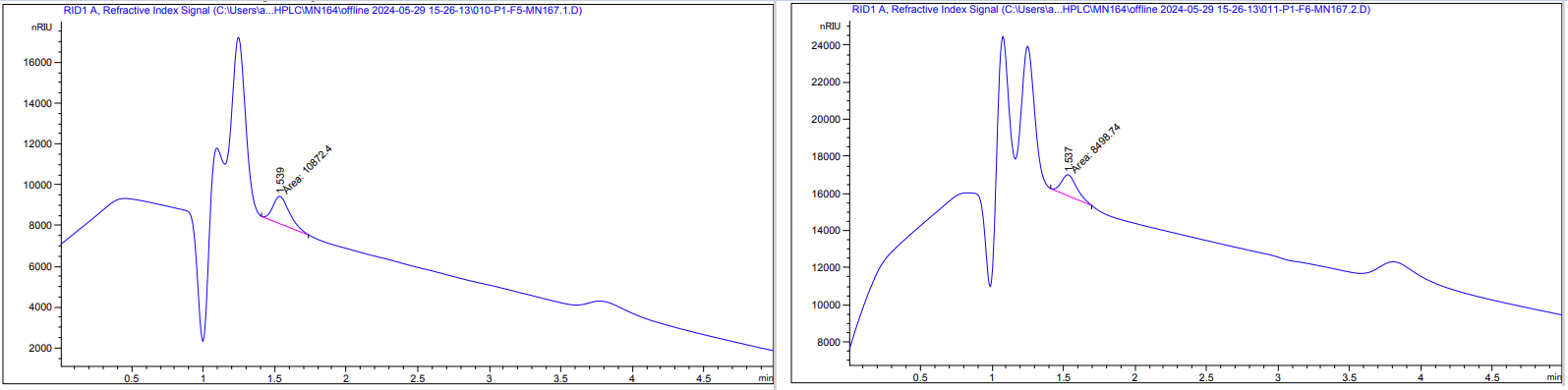


| **Figure S2:** *HPLC graphs of formic acid detection.* |
| --- |

## Crystal structures and crystallographic data

| 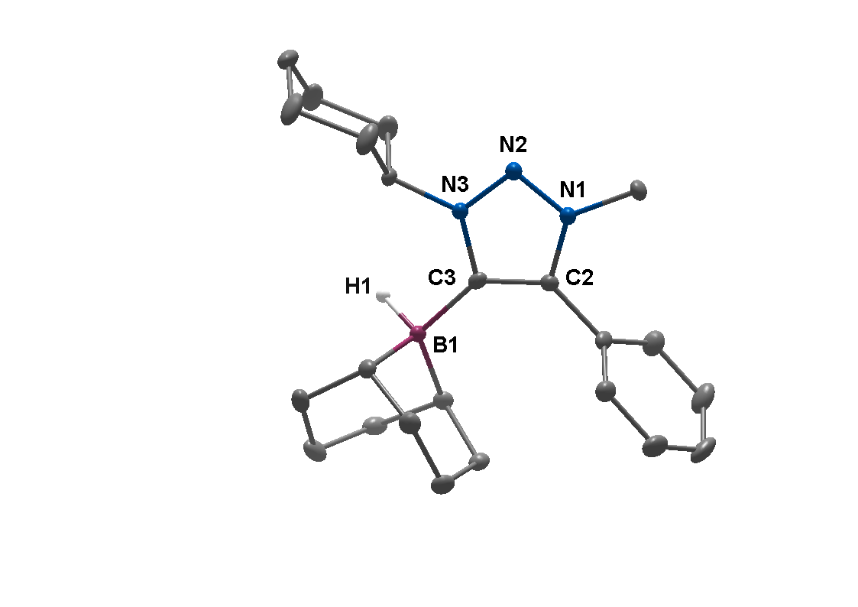 | | | |
| --- | --- | --- | --- |
| **Figure S3:** *ORTEP representation of* ***B1****: ellipsoids drawn at 50% probability. Solvent molecules and H‑atoms omitted for clarity.* | | | |
| **Table S1:** *Selected bond lengths and bond angles of* ***B1****.* | | | |
| Atom | Bond length (Å) | Atom | Bond angle (°) |
| C2–C3 | 1.391(2) | C2–C3–N3 | 101.8(1) |
| C3–N3 | 1.376(2) | C3–N3–N2 | 115.3(1) |
| N3–N2 | 1.328(2) | N3–N2–N1 | 103.1(1) |
| N2–N1 | 1.317(2) | N2–N1–C2 | 112.8(1) |
| N1–C2 | 1.364(2) | N1–C2–C3 | 107.0(1) |
| C3–B1 | 1.634(2) | B1–C3–N3 | 120.2(1) |
|  |  | B1–C3–C2 | 138.0(1) |

| 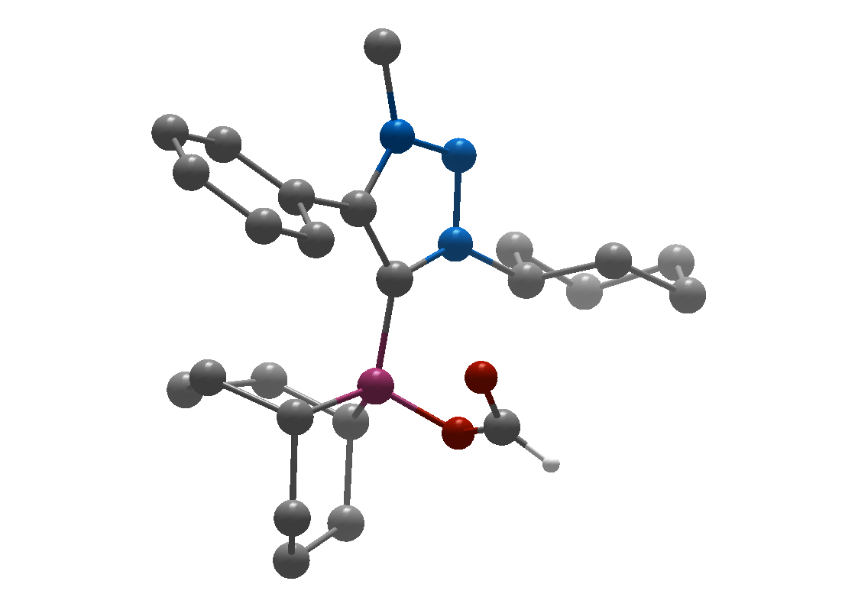 |
| --- |
| **Figure S4:** *Ball and stick model of* ***B2*** *via X-ray diffraction. The quality of the collected data was not sufficient enough to*  *acquire a structure.* |

| 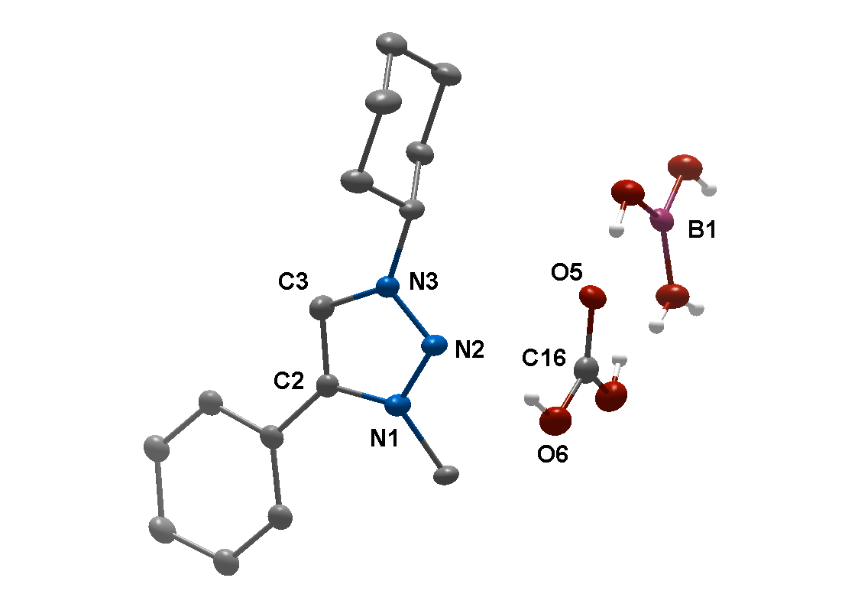 | | | |
| --- | --- | --- | --- |
| **Figure S5:** *ORTEP representation of* ***4****: ellipsoids drawn at 50% probability. Solvent molecules and H‑atoms omitted for clarity.* | | | |
| **Table S2:** *Selected bond lengths and bond angles of* ***4****.* | | | |
| Atom | Bond length (Å) | Atom | Bond angle (°) |
| N1–N2 | 1.327(3) | N1–N2–N3 | 104.7(2) |
| N2–N3 | 1.309(3) | N2–N3–C3 | 112.1(2) |
| N3–C3 | 1.352(3) | N3–C3–C2 | 106.7(2) |
| C3–C2 | 1.370(4) | C3–C2–N1 | 103.9(2) |
| C2–N1 | 1.365(3) | C2–N1–N2 | 112.6(2) |
| C16–O5 | 1.300(3) | O6–C16–O5 | 122.0(1) |
| C16–O6 | 1.317(2) |  |  |

| 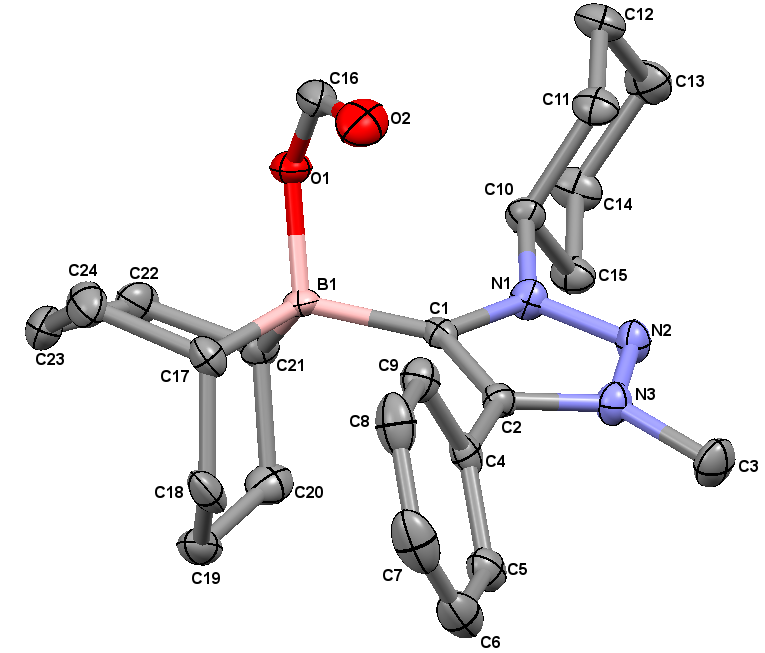 |
| --- |
| **Figure S6:** *ORTEP representation of* ***B2*** *ellipsoids drawn at 50% probability. Solvent molecules and H‑atoms omitted for clarity.* |

| **Table S3:** *Selected bond lengths and bond angles of* ***B2****.* | | | |
| --- | --- | --- | --- |
| Atom | Bond length (Å) | Atom | Bond angle (°) |
| O1-C16 | 1.298(5) | C16-O1-B1 | 123.4(4) |
| O1-B1 | 1.551(6) | O2-C16-O1 | 127.9(4) |
| O2-C16 | 1.208(5) | O1-B1-C1 | 107.0(3) |
| C1-B1 | 1.644(6) | O1-B1-C17 | 111.3(4) |
| C17-B1 | 1.621(6) | O1-B1-C21 | 104.9(4 |
| C21-B1 | 1.630(6) | N1-C1-B1 | 122.7(3) |
|  |  | N1-C1-C2 | 102.2(3) |
|  |  | N3-N2-N1 | 103.4(3) |

**Table S4:** *Crystallographic details.*

|  | **B1** | **B2** |
| --- | --- | --- |
| Chemical formula | C_23_H_34_BN_3_ | C_24_H_34_BN_3_O_2_ |
| *M*_r_ | 363.34 | 407.35 |
| Crystal system  Space group | Monoclinic  *P*2(1)/*c* | Orthorhombic  *P2c/2n* |
| a (Å)  b (Å)  c (Å)  α (°)  β (°)  γ (°) | 12.0407(5)  10.7221(5)  15.8109(7)  90  93.616(2)  90 | 17.9188(14)  9.0564(7)  27.260(2)  90  90  90 |
| V (Å^3^) | 2037.15(16) | 4423.7(6) |
| Z | 4 | 8 |
| Densitiy (g cm^-3^) | 1.185 | 1.223 |
| F(000) | 792 | 1760 |
| Radiation Type | MoK_α_ | MoK_α_ |
| μ (mm^-1^) | 0.069 | 0.077 |
| Crystal size | 0.3x0.26x0.14 | 0.32 x 0.19 x 0.11 |
| Meas. Refl. | 22782 | 18728 |
| Indep. Refl. | 3733 | 8905 |
| Obsvd. [*I* > 2σ(*I*)] refl. | 3136 | 5960 |
| R_int_ | 0.0470 | 0.0477 |
| R [F^2^ > 2σ(F^2^)], wR(F^2^), S | 0.0434, 0.1070, 0.0544 | 0.0533, 0.1060, 0.993 |
| Δρ_max_, Δρ_min_ (e Å^-3^)  CCDC | 2052661 2379971 | 0.243, -0.232 |

**Table S5:** *Crystallographic details.*

|  | **4** |
| --- | --- |
| Chemical formula | C_16_H_24_BN_3_O_6_ |
| *M*_r_ | 365.19 |
| Crystal system  Space group | Orthorhombic  *P* 21/*n* 21/*m* 21/*a* |
| a (Å)  b (Å)  c (Å)  α (°)  β (°)  γ (°) | 15.3947(3)  6.84400(10)  16.4402(3)  90  90  90 |
| V (Å^3^) | 1732.16(5) |
| Z | 4 |
| Densitiy (g cm^-3^) | 1.400 |
| F(000) | 776 |
| Radiation Type | CuK_a_ |
| μ (mm^-1^) | 0.886 |
| Crystal size | 0.36x0.17x0.08 |
| Meas. Refl. | 19112 |
| Indep. Refl. | 1855 |
| Obsvd. [*I* > 2σ(*I*)] refl. | 1548 |
| R_int_ | 0.0483 |
| R [F^2^ > 2σ(F^2^)], wR(F^2^), S | 0.0477, 0.1258, 1.096 |
| Δρ_max_, Δρ_min_ (e Å^-3^)  CCDC | 0.226, –0.304  2052662 |

## Cyclic voltammetry

| 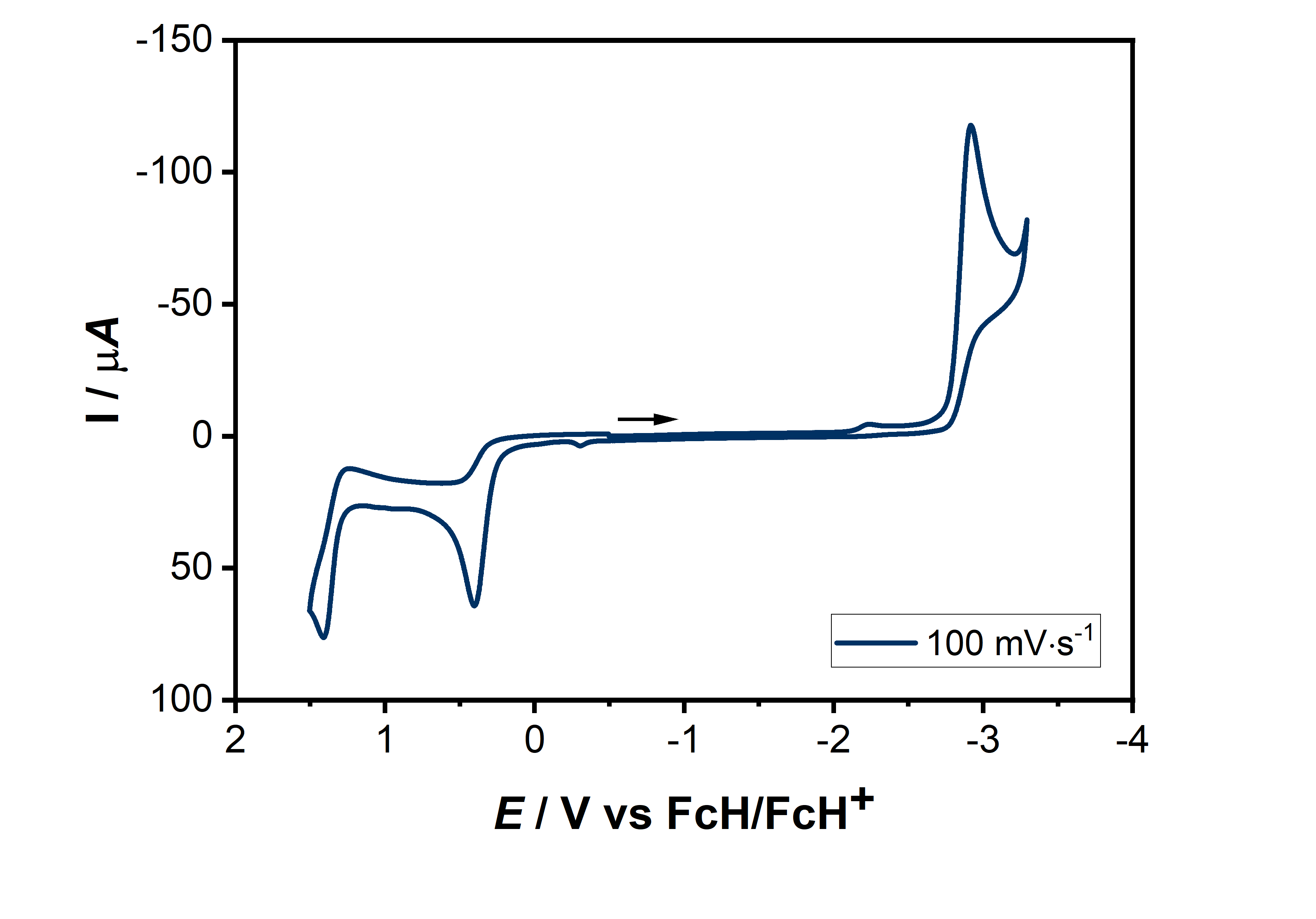 | 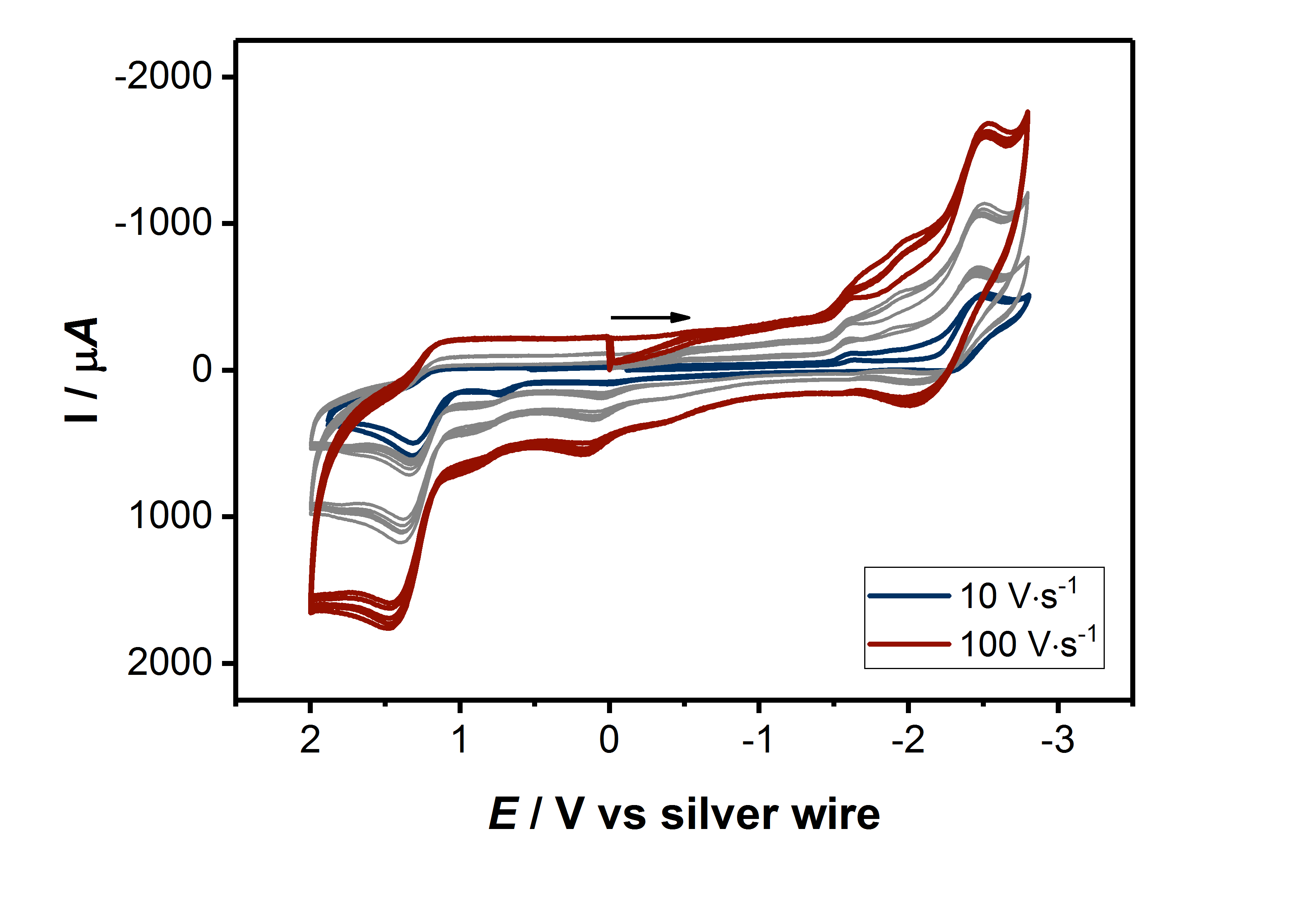 |
| --- | --- |
| 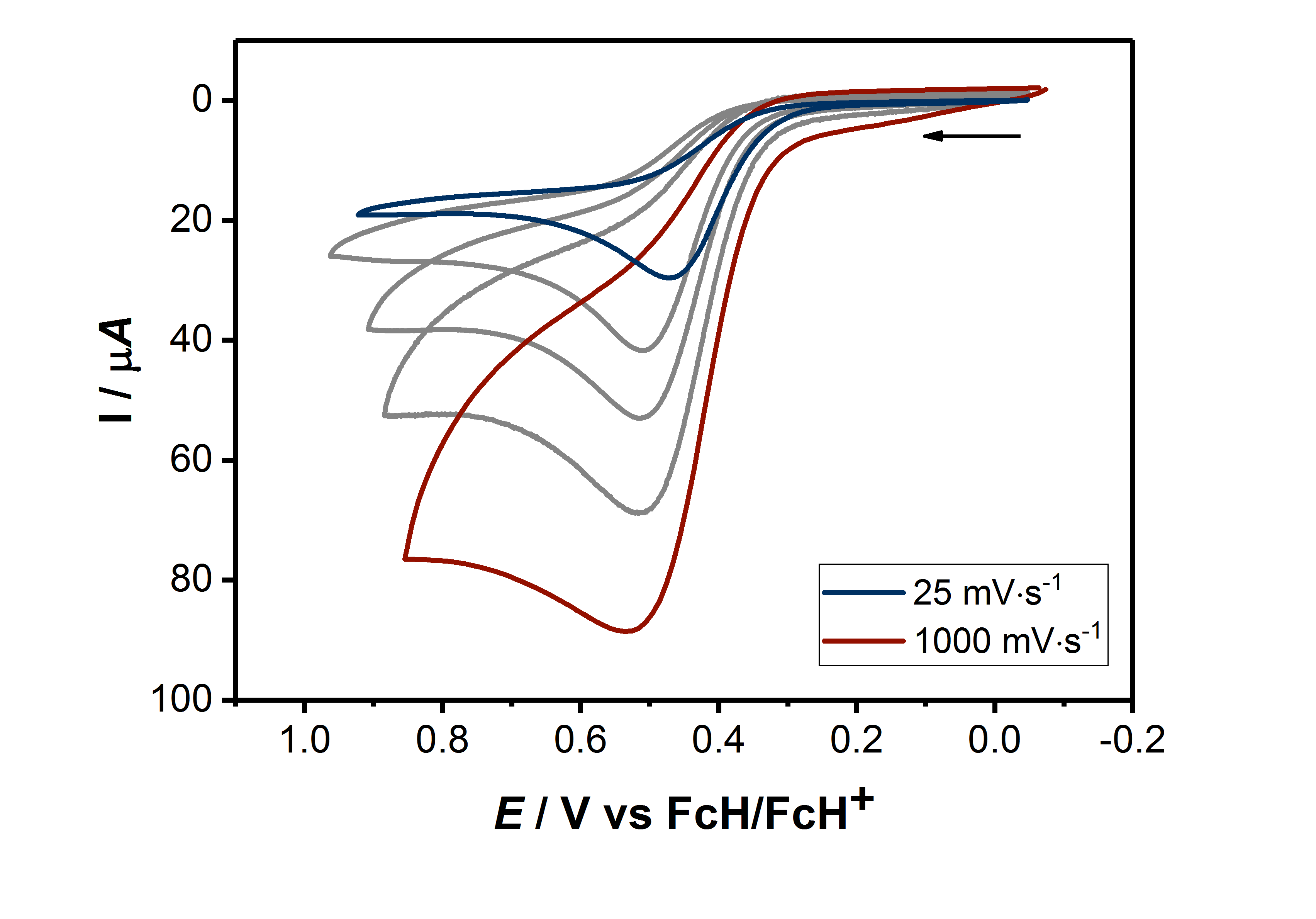 | 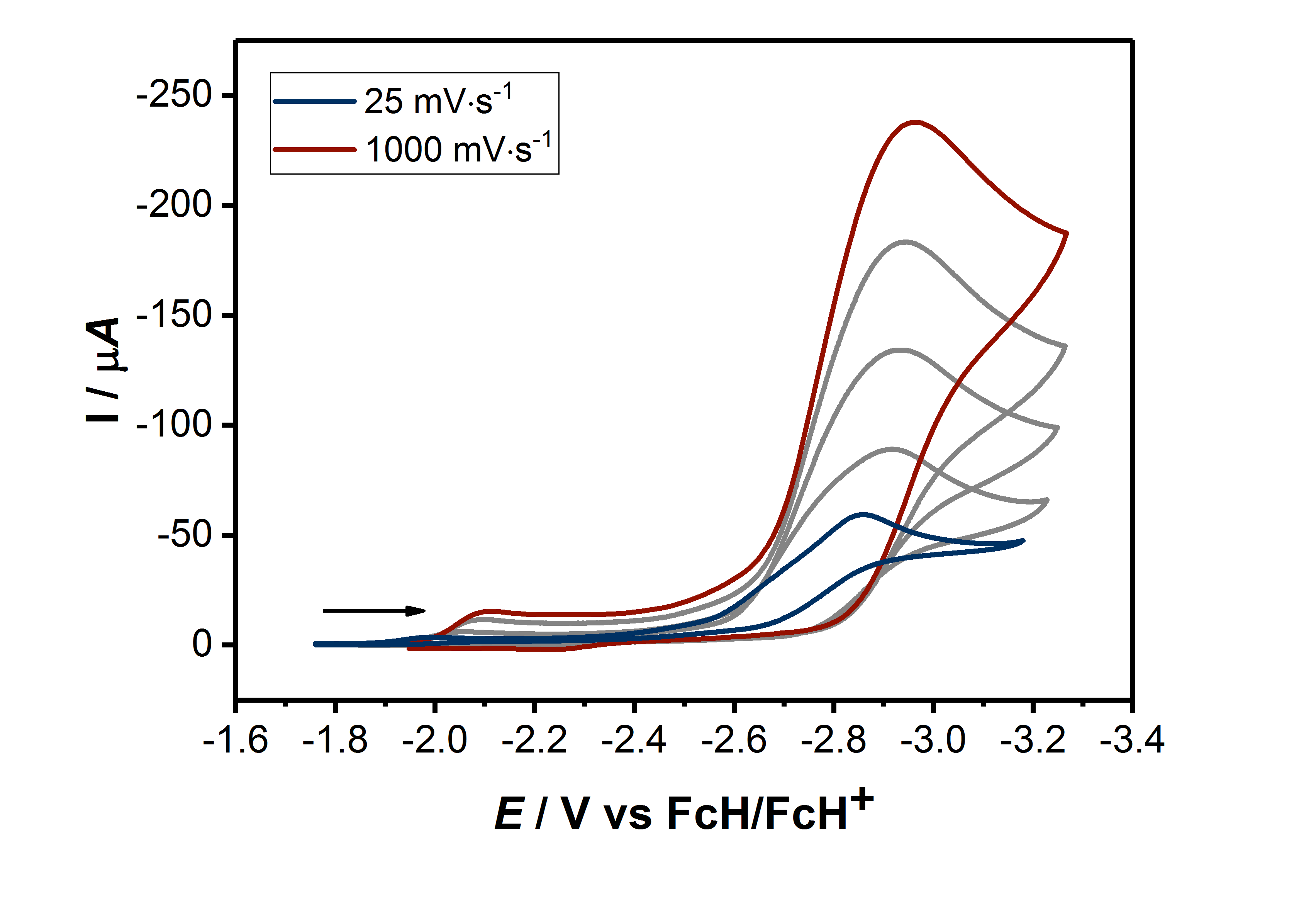 |
| **Figure S7:** *Cyclic voltammograms of a 0.1 mM solution of* ***B1*** *with 0.1 M NBu_4_PF_6_ in MeCN at different scan rates.* | |

## NMR Spectroscopy


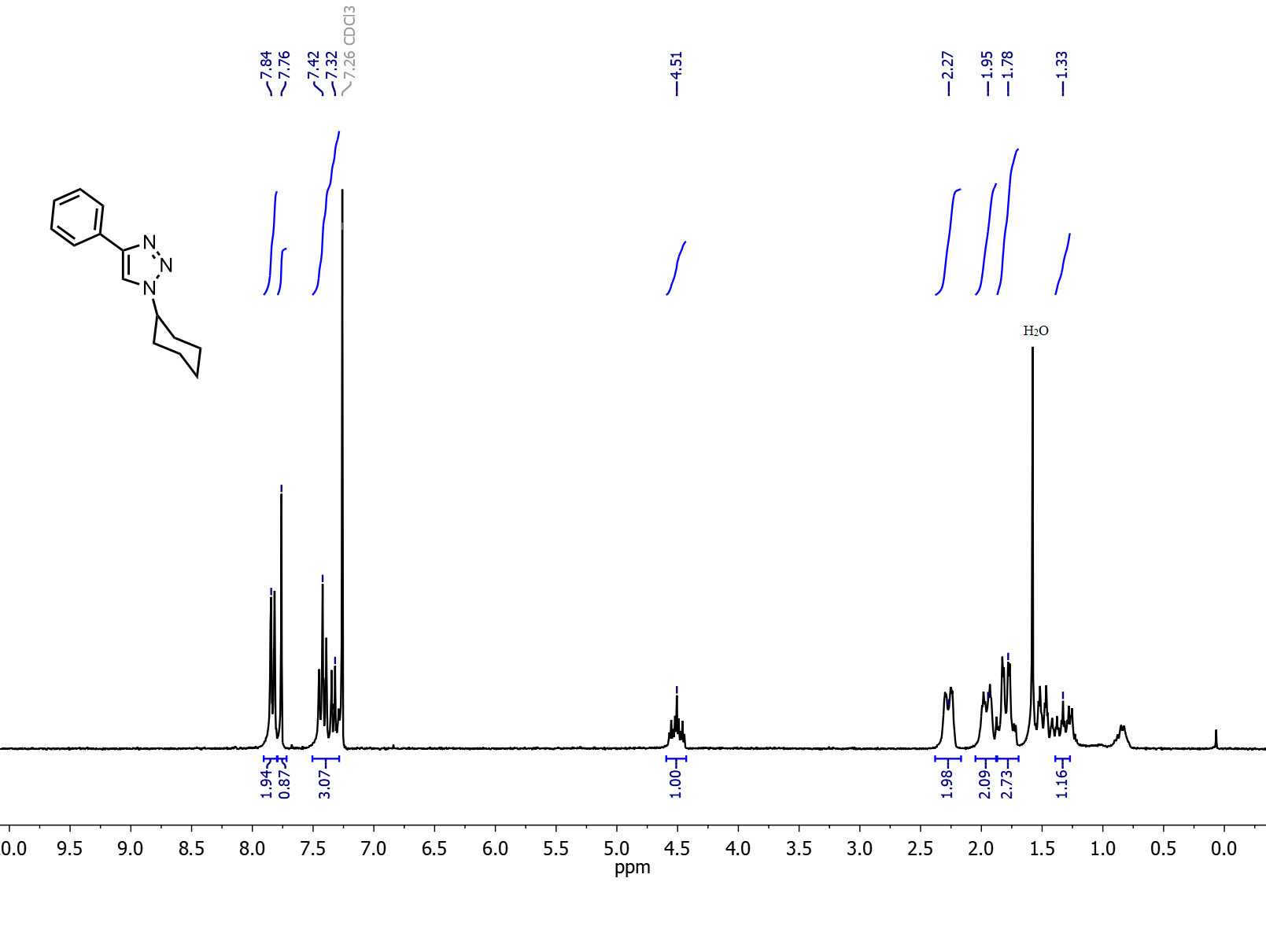


**Figure S8:** *^1^H NMR of* ***1*** *in CDCl_3_.*

*
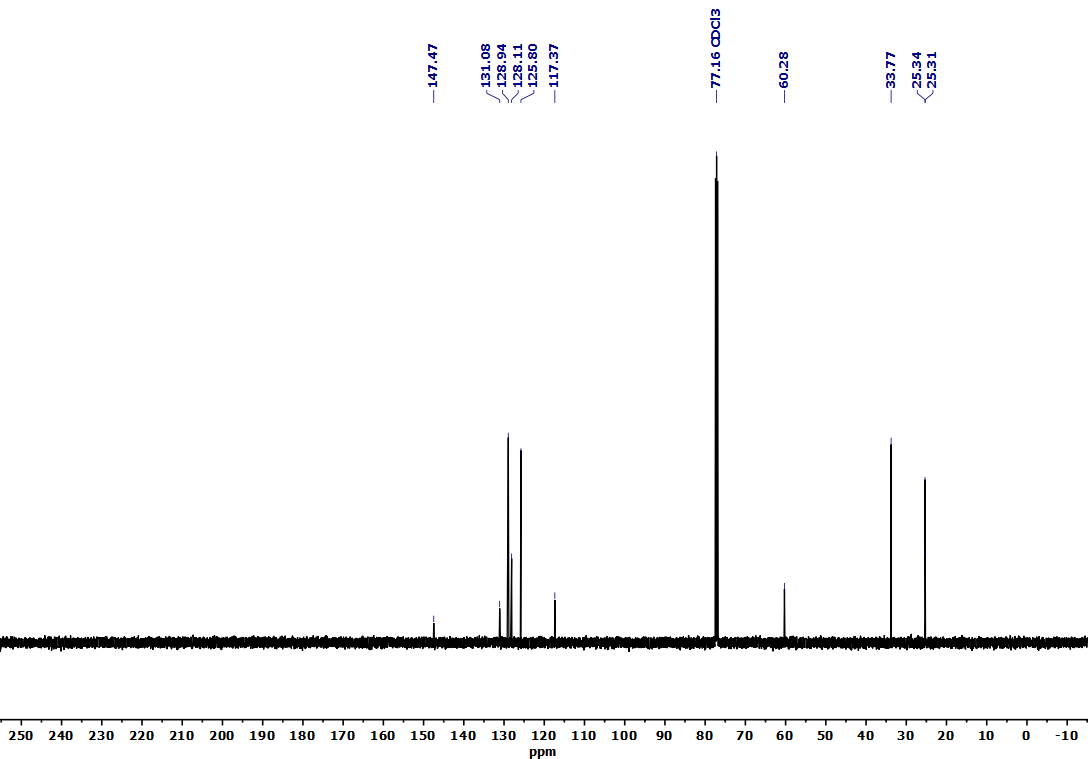
*

**Figure S9:** *^13^C NMR of* ***1*** *in CDCl_3_.*


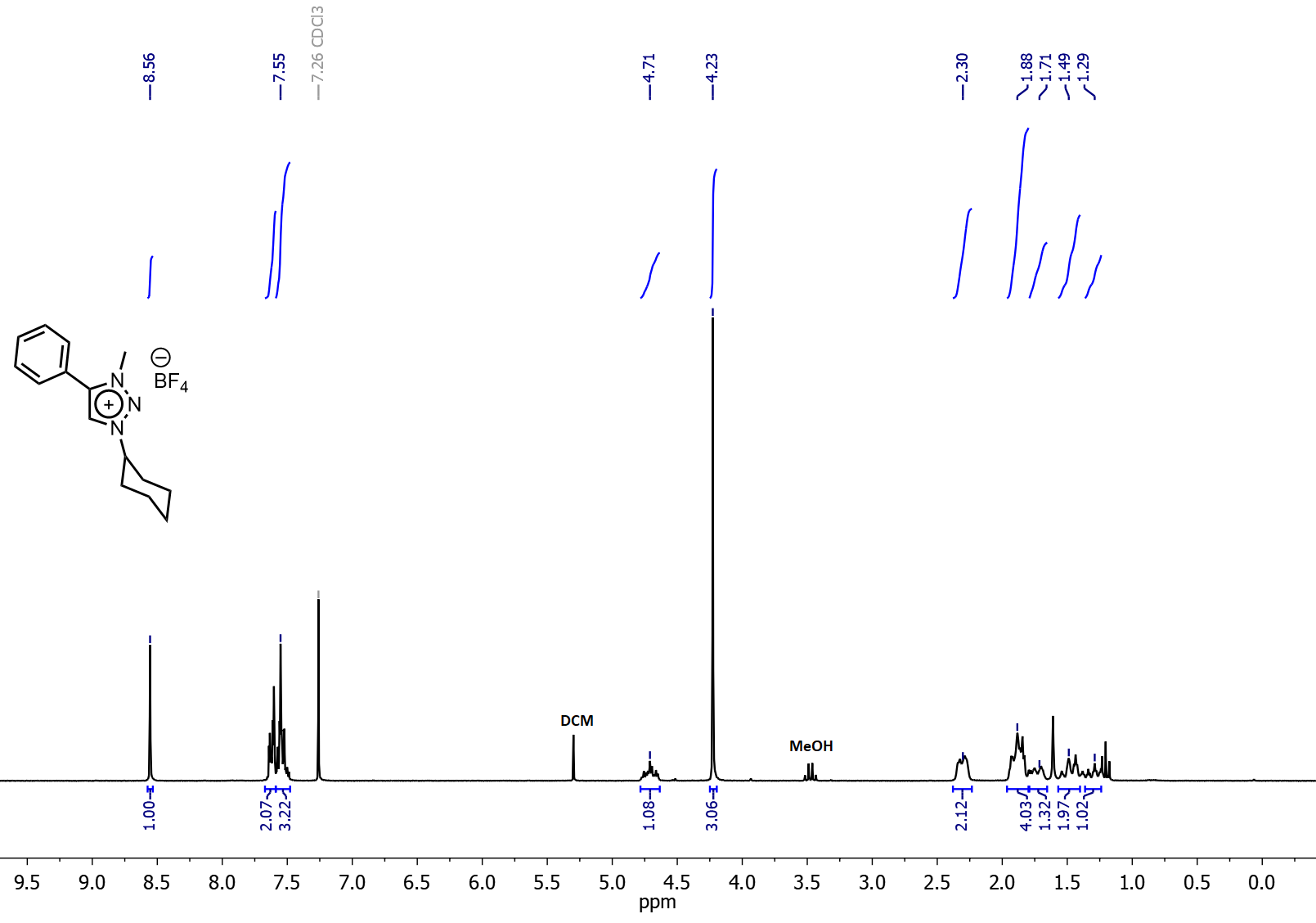


**Figure S10:** *^1^H NMR of* ***2*** *in CDCl_3_.*


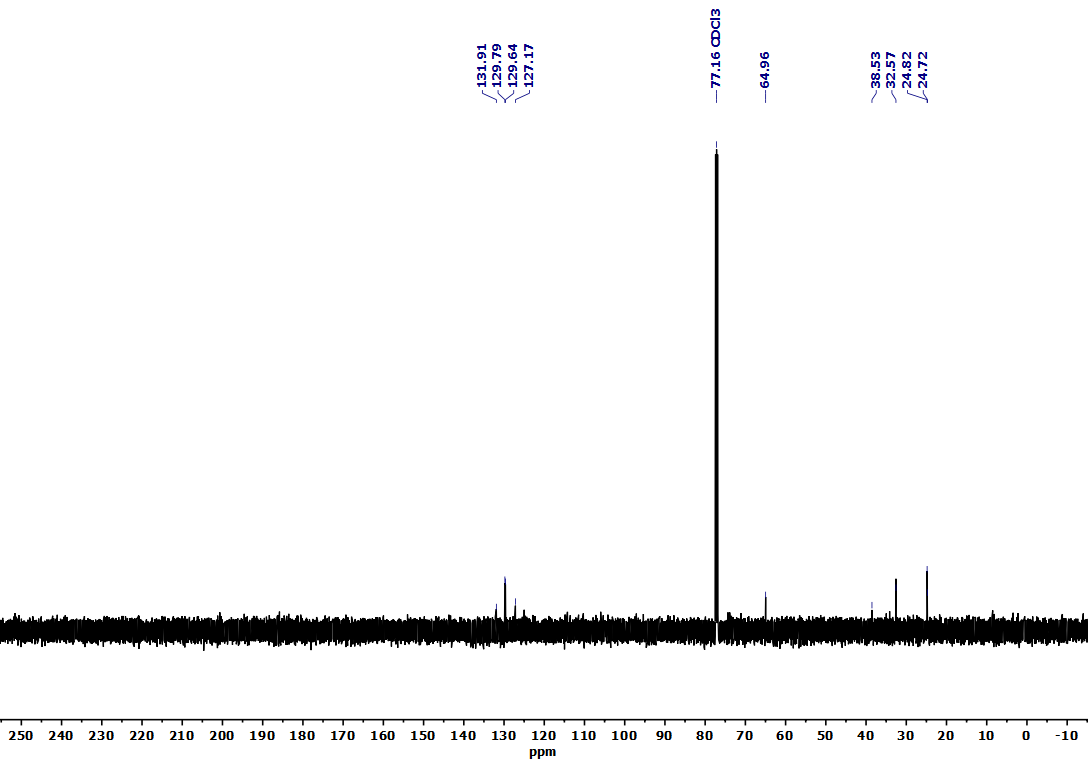


**Figure S11:** *^13^C NMR of* ***2*** *in CDCl_3_.*


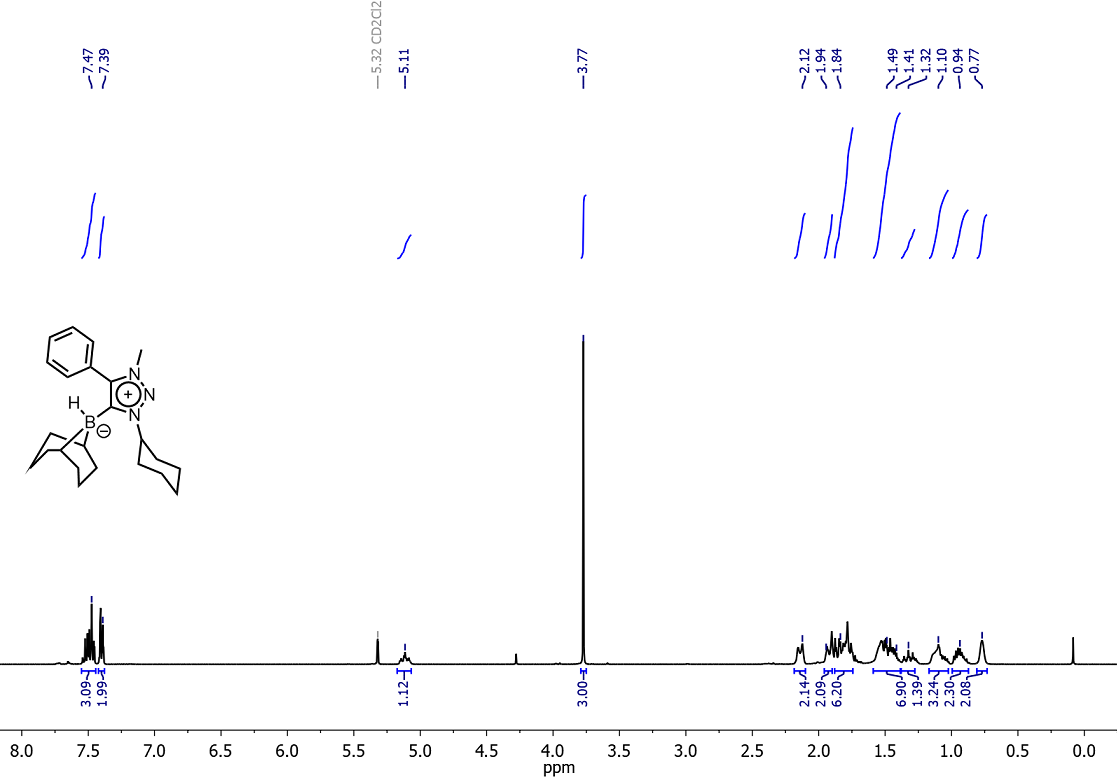


**Figure S12:** *^1^H NMR of* ***B1*** *in CD_2_Cl_2_.*


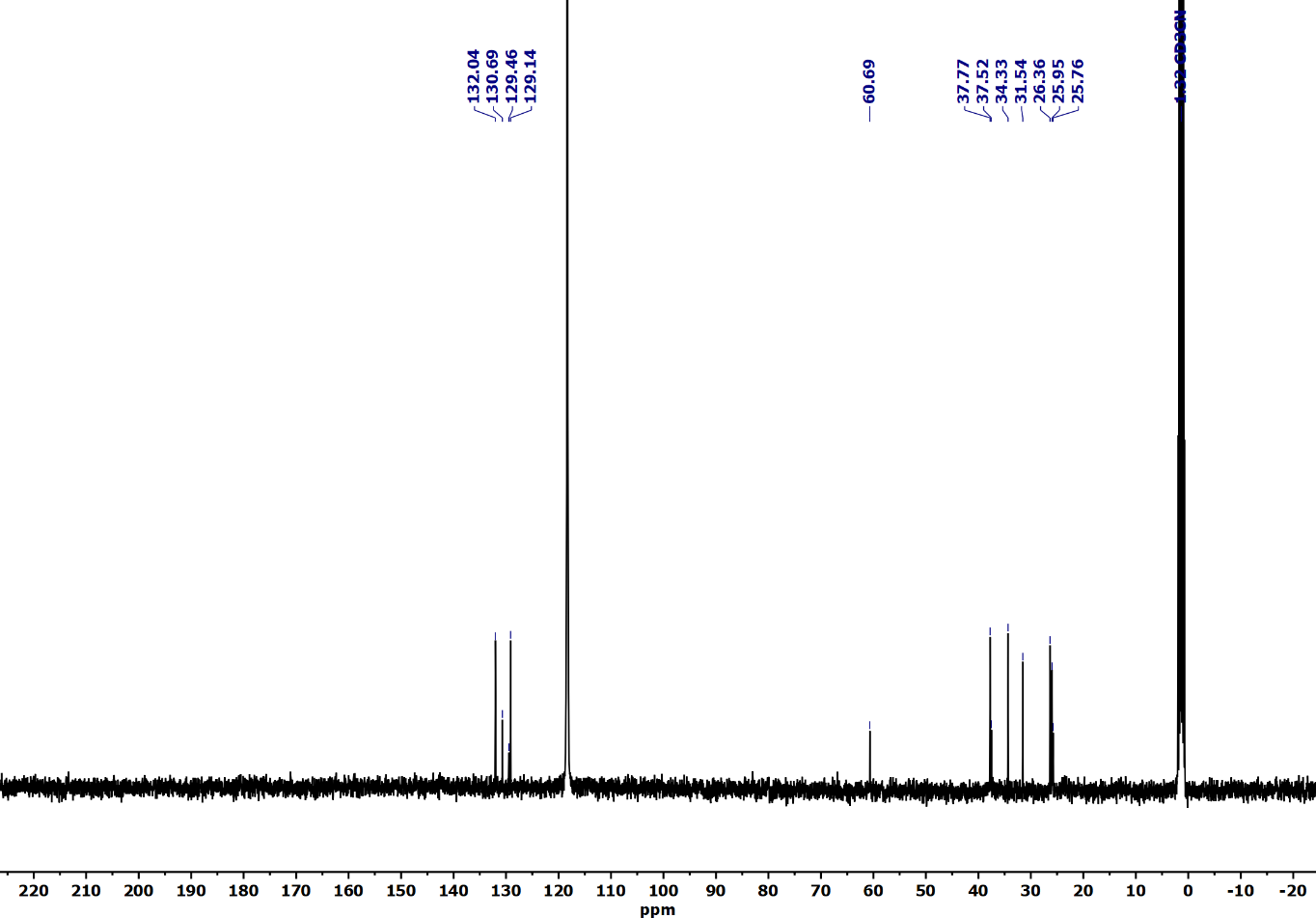


**Figure S13:** *^13^C NMR of* ***B1*** *in CD_2_Cl_2_.*

**Figure S14:** *^11^B NMR of* ***B1*** *in CD_2_Cl_2_.*


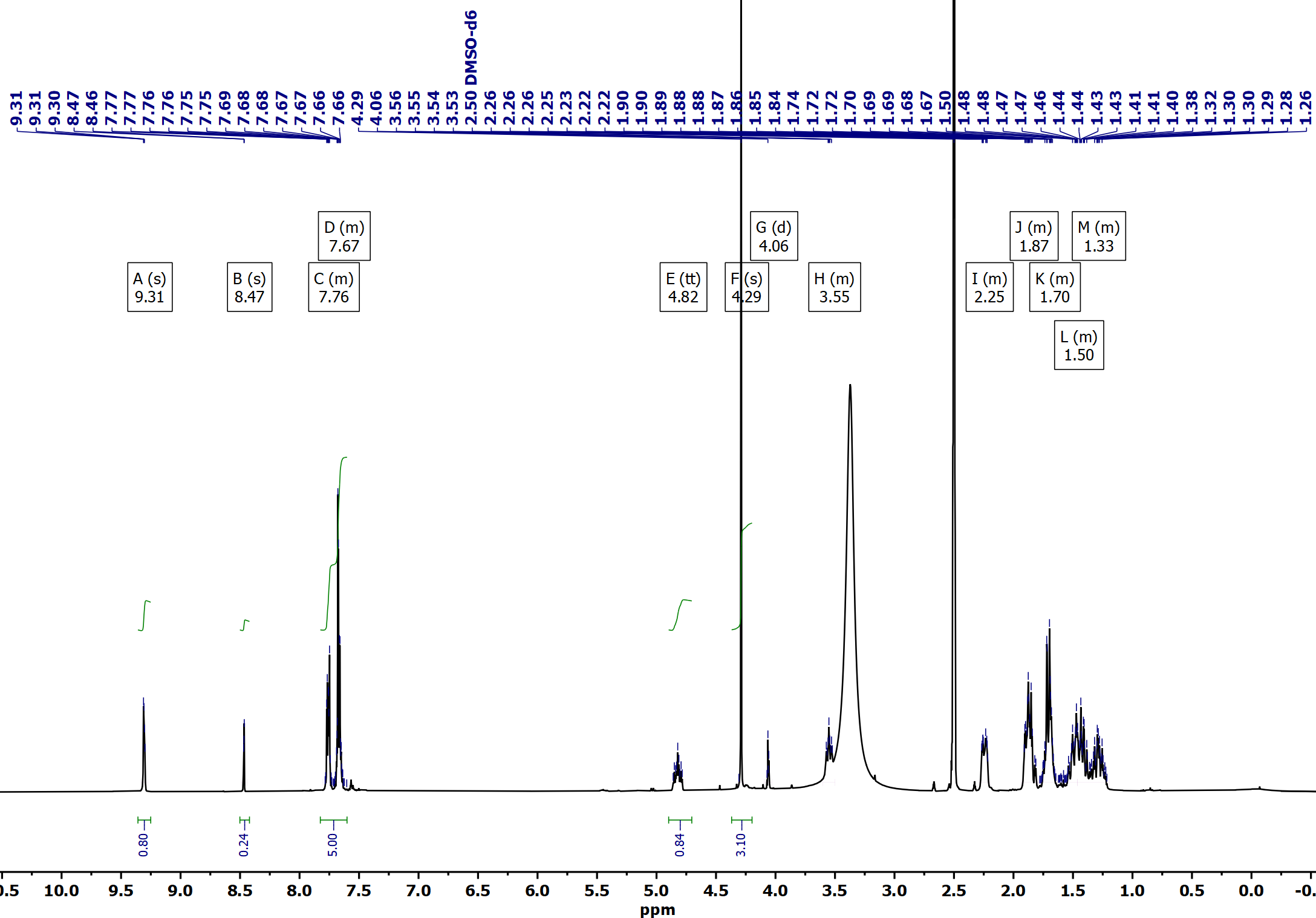


**Figure S15:** *^1^H NMR of* ***4*** *in DMSO-d_6_.*


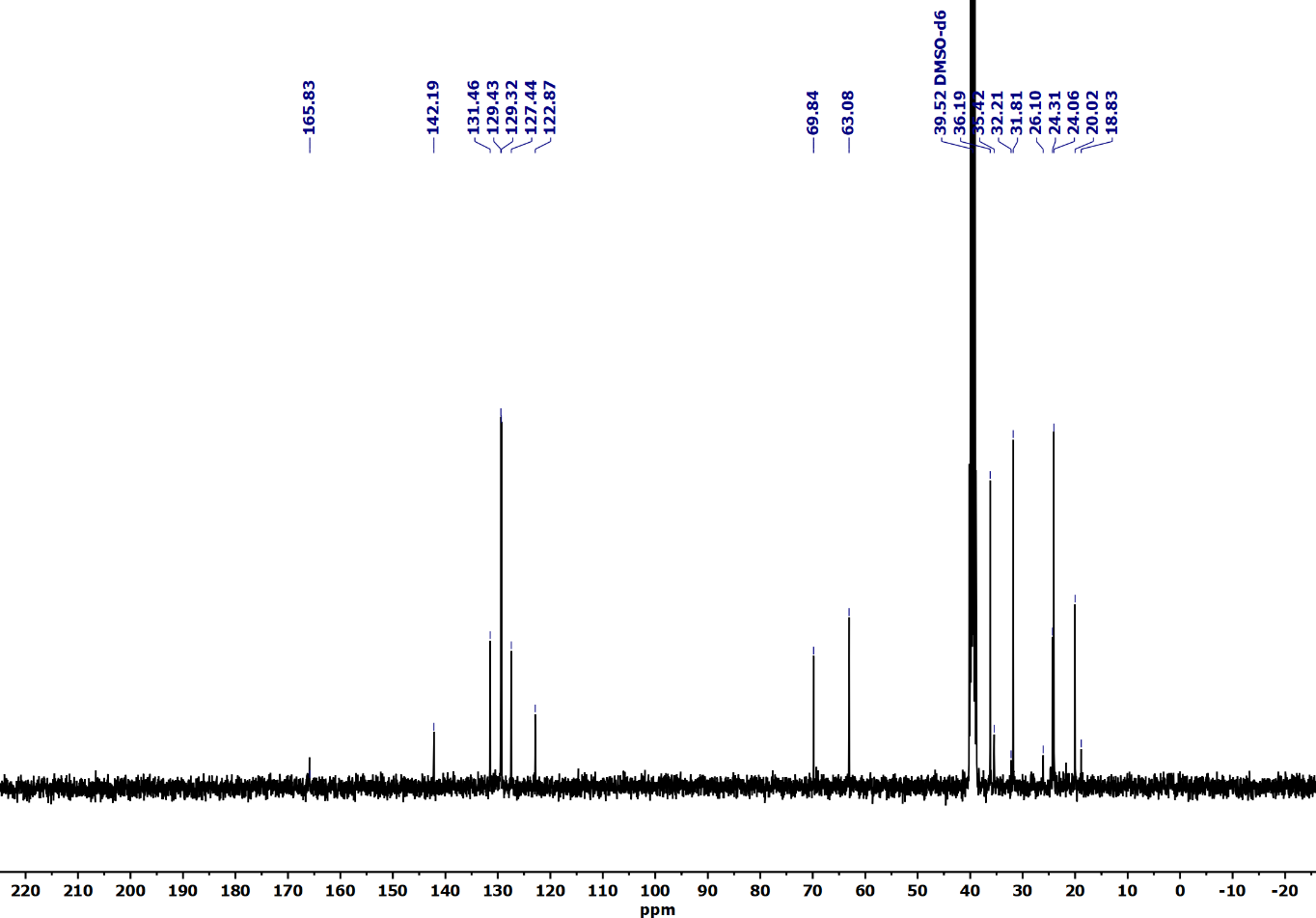


**Figure S16:** *^13^C NMR of* ***4*** *in DMSO-d_6_.*


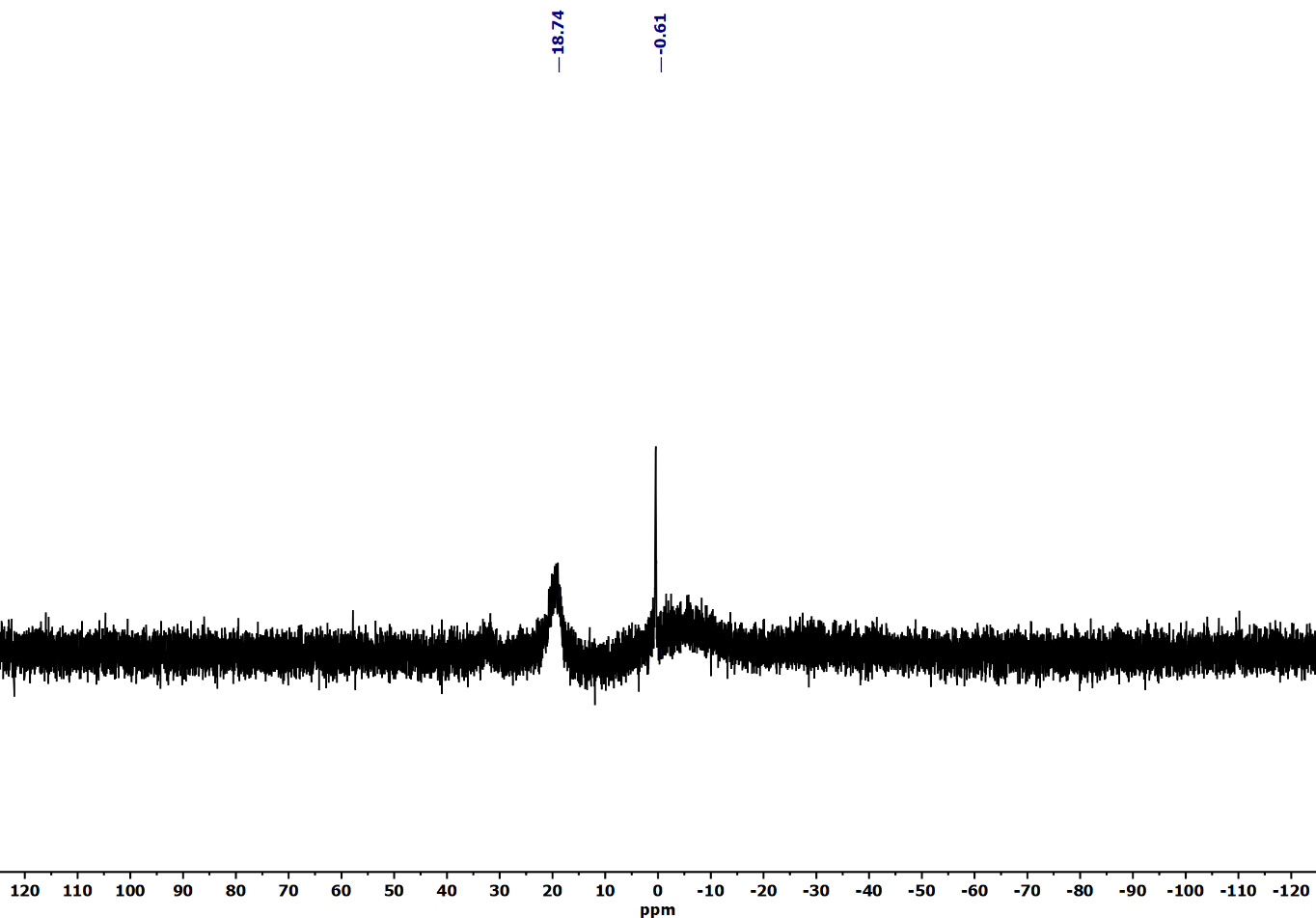


**Figure S17:** *^11^B NMR of* ***4*** *in DMSO-d_6_.*


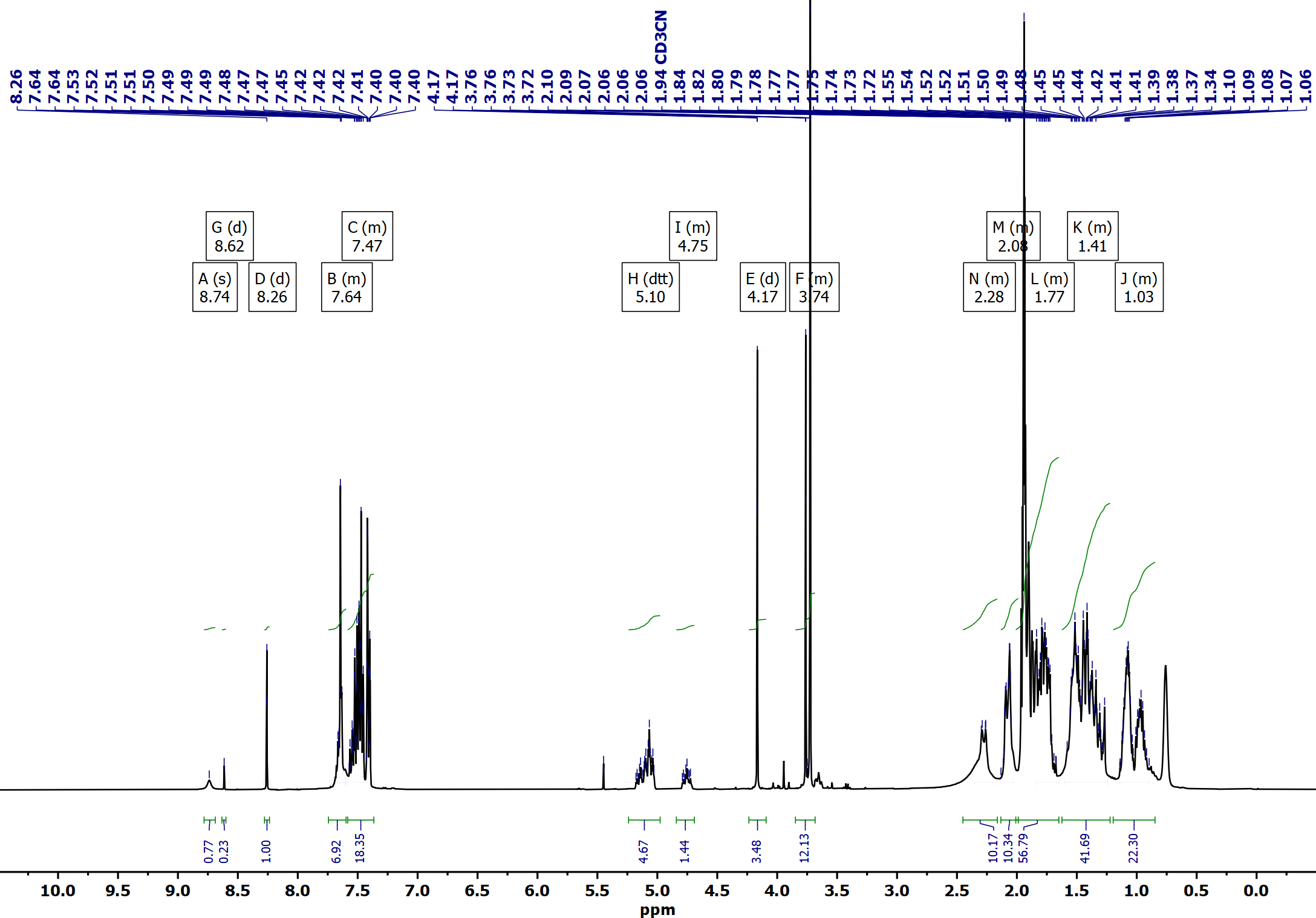


**Figure S18:** *^1^H NMR of crude reaction mixture in the* reaction of **B1** with atmospheric CO_2_ in CD_3_CN.


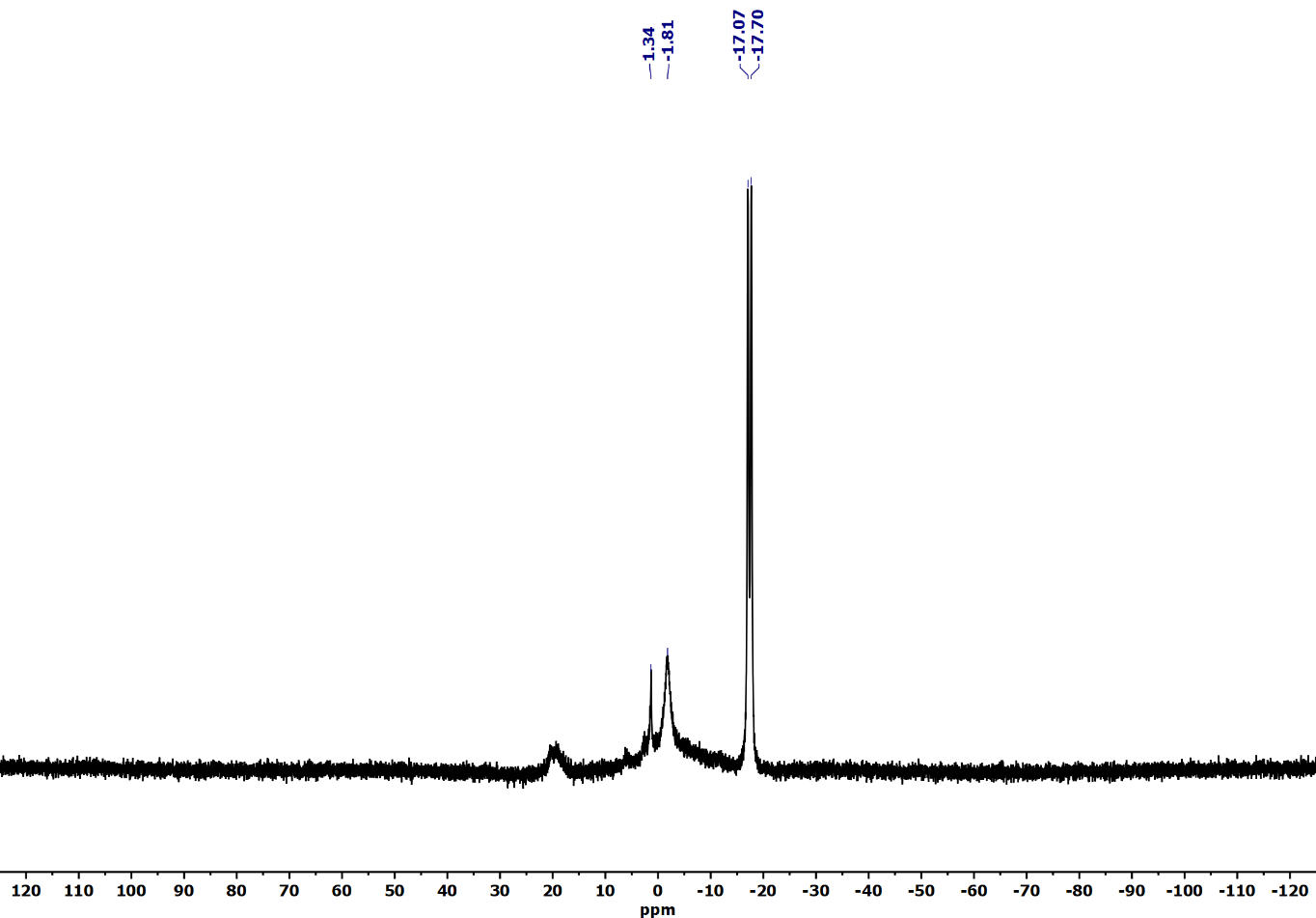


**Figure S19:** *^11^B NMR of crude reaction mixture in the reaction of* ***B1*** *with atmospheric CO_2_ in CD_3_CN.*


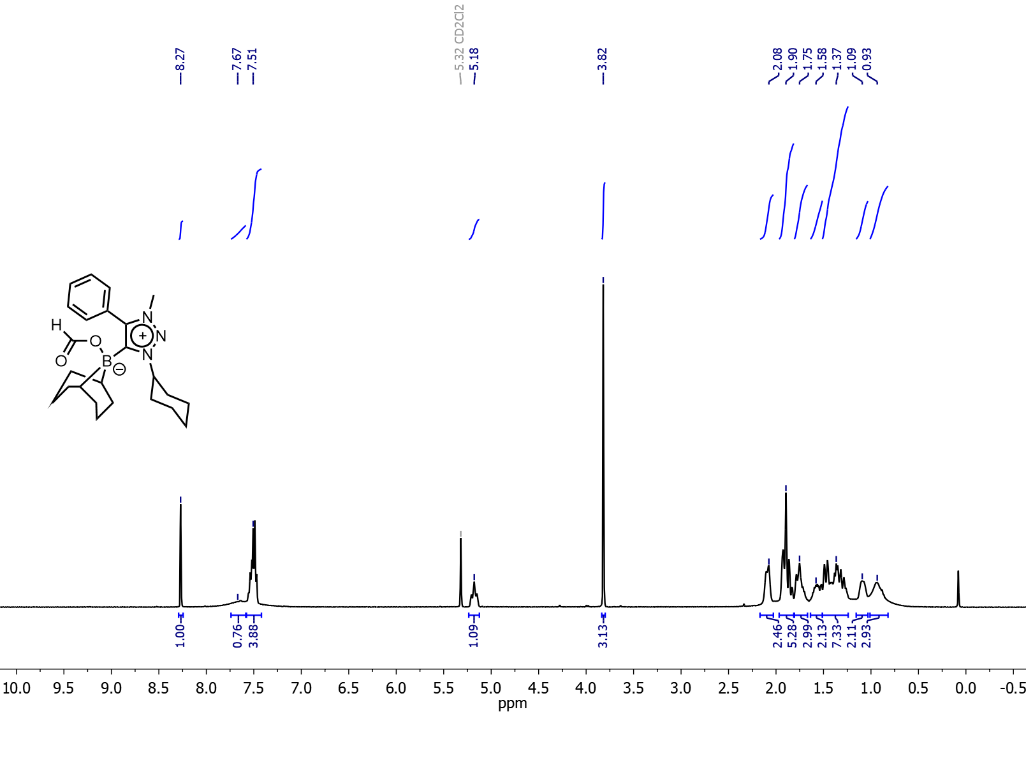


**Figure S20:** *^1^H NMR of* ***B2*** *in CD_2_Cl_2_.*


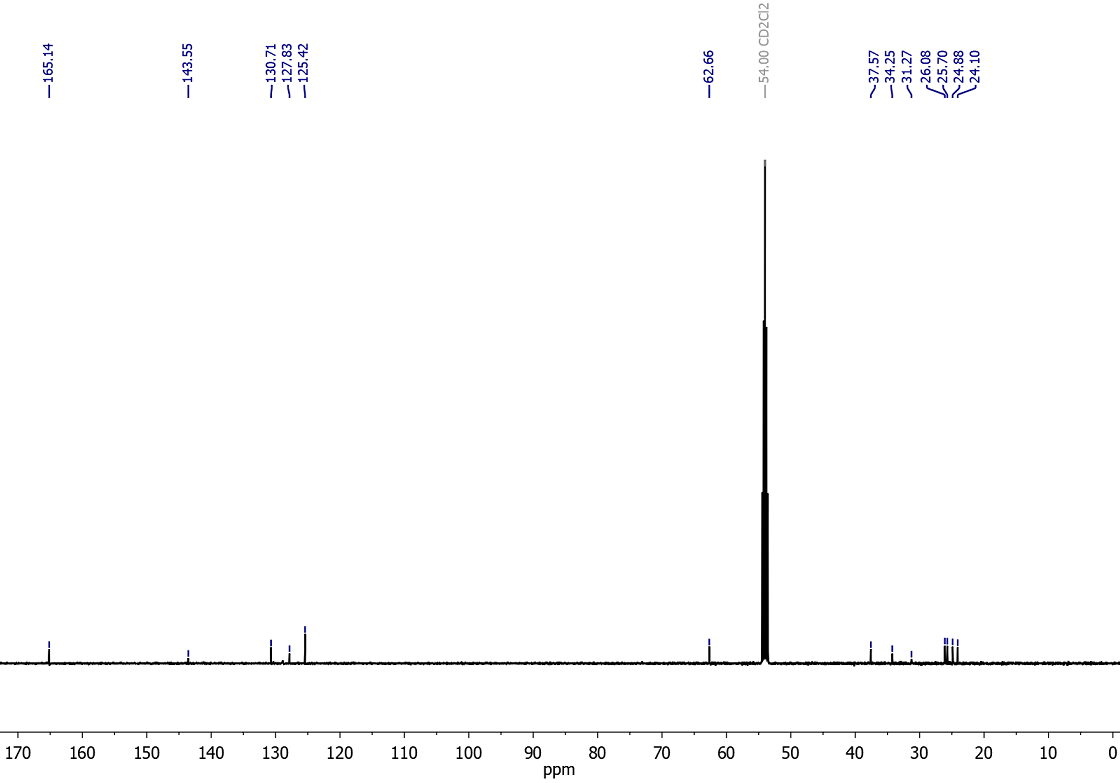


**Figure S21:** *^13^C NMR of* ***B2*** *in CD_2_Cl_2_.*

*
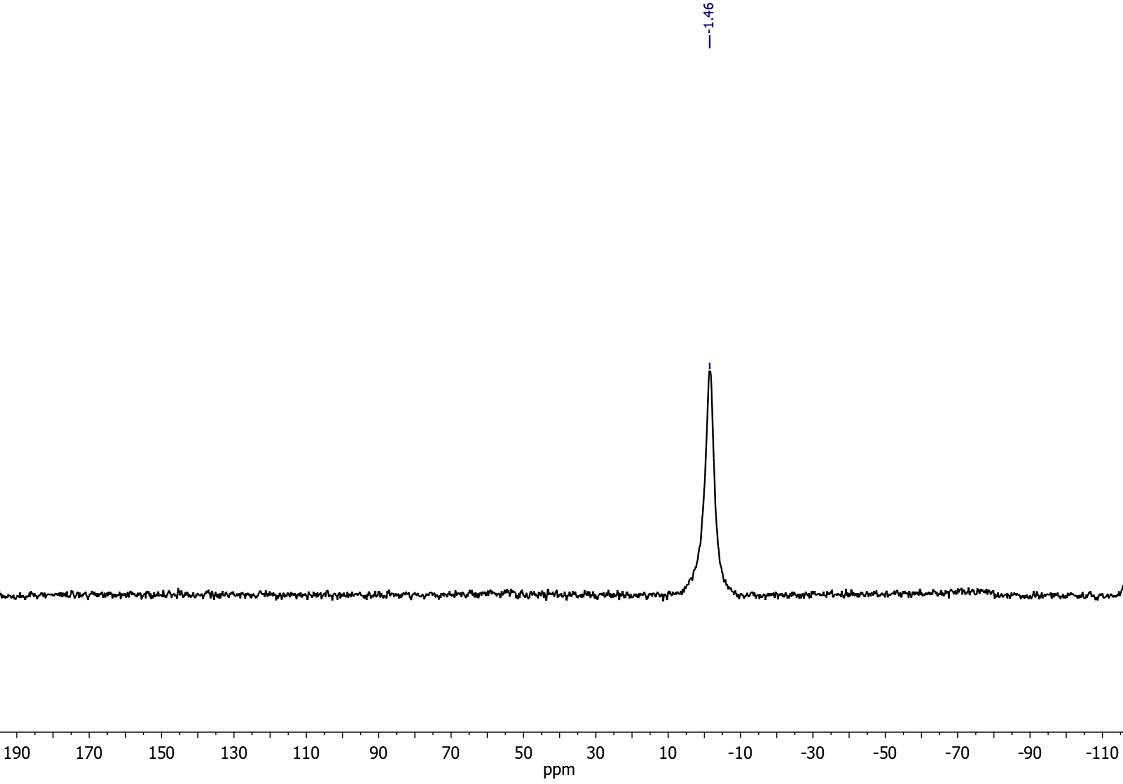
*

**Figure S22:** *^11^B NMR of* ***B2*** *in CD_2_Cl_2_.*

**Figure S23:** *^1^H NMR of crude reaction mixture in the synthesis of* ***B2 with Amminborane*** *in CD_2_Cl_2_.*

**Figure S24:** *^11^B NMR of of crude reaction mixture in the synthesis of* ***B2 with Amminborane*** *in CD_2_Cl_2_.*

**Figure S25:** *^1^H NMR of* ***formic acid mixture*** *in D_2_O.*

## References

[1] G. R. Fulmer, A. J. M. Miller, N. H. Sherden, H. E. Gottlieb, A. Nudelman, B. M. Stoltz, J. E. Bercaw, K. I. Goldberg, *Organometallics* **2010**, *29*, 2176–2179.

[2] A. L. Spek, *J. Appl. Crystallogr.* **2003**, *36*, 7–13.

[3] G. M. Sheldrick, *Acta Crystallogr., Sect. C: Struct. Chem.* **2015**, *71*, 3–8.

[4] G. M. Sheldrick, *Acta Crystallogr., Sect. A: Found. Crystallogr.* **2008**, *64*, 112–122.

[5] G. M. Sheldrick, *SHELXS-97, Program for Crystal Structure Solution and Refinement*, University of Göttingen, Göttingen, Germany, **1997**.

[6] G. M. Sheldrick, *SHELXL Version 2014/7, Program for Chrystal Structure Solution and Refinement*, University of Göttingen, Germany, **2014**.

[7] G. M. Sheldrick, *SADABS Ver. 2008/1, SADABS. Program for Empirical Absorption Correction*, University of Gottingen, Germany, **2012**.

[8] SAINT+, *Data Integration Engine, Version 8.27b©*, Bruker AXS Inc., Madison, Wisconsin, USA, **1997-2012**.

[9] O. V. Dolomanov, L. J. Bourhis, R. J. Gildea, J. A. K. Howard, H. Puschmann, *J. Appl. Crystallogr.* **2009**, *42*, 339–341.

[10] K. Asano, S. Matsubara, *Org. Lett.* **2010**, *12*, 4988–4991.

[11] Ashley M. King, Hazel A. Sparkes, Richard L. Wingad, and Duncan F. Wass, *Organometallics* **2020** *39*, 3873-3878

[12] Y. Wang, Z. Shao, K. Zhang, Q. Liu, *Angew. Chem.* **2018**, *130*, 15363.
